# E2F1-initiated transcription of PRSS22 promotes breast cancer metastasis by cleaving ANXA1 and activating FPR2/ERK signaling pathway

**DOI:** 10.1038/s41419-022-05414-3

**Published:** 2022-11-21

**Authors:** Lin Song, Hui Li, Ran-Ran Ma, Sen Liu, Guo-Hao Zhang, Xiang-Yu Guo, Rui-Nan Zhao, Xiao-Juan Wu, Kai Zhang, Peng Gao

**Affiliations:** 1 Department of Pathology, Qilu Hospital of Shandong University, Jinan, Shandong 250012 China; 2 Department of Pathology, Shandong Provincial Hospital Affiliated to Shandong First Medical University, Jinan, Shandong 250021 China; 3 Key Laboratory for Experimental Teratology of the Ministry of Education and Department of Pathology, School of Basic Medical Sciences, Cheeloo College of Medicine, Shandong University, Jinan, Shandong 250012 China; 4 Department of Breast Surgery, Qilu Hospital of Shandong University, Jinan, Shandong 250012 China

**Keywords:** Breast cancer, Oncogenesis, Cell invasion

## Abstract

Breast cancer (BC) is the most common malignant tumor in women worldwide. Metastasis is the main cause of BC-related death. The specific mechanism underlying BC metastasis remains obscure. Recently, PRSS22 was discovered to be involved in tumor development, however, its detailed biological function and regulatory mechanism in BC are unclear. Here, we characterized that PRSS22 expression is upregulated in BC tissues compared with non-tumorous breast tissues. Dual luciferase assays, bioinformatics analyses and chromatin immunoprecipitation (ChIP) assays indicated that transcription factor E2F1 directly binds to the PRSS22 promoter region and activates its transcription. Functionally, upregulation of PRSS22 promoted invasion and metastasis of BC cells in vitro and in vivo, whereas knockdown of PRSS22 inhibited its function. Mechanistically, the combination of PRSS22 and ANXA1 protein in BC cells was first screened by protein mass spectrometry analysis, and then confirmed by co-immunoprecipitation (Co-IP) and western blot assays. Co-overexpression of PRSS22 and ANXA1 could promote BC cell migration and invasion. We further demonstrated that PRSS22 promotes the cleavage of ANXA1 and in turn generates an N-terminal peptide, which initiates the FPR2/ERK signaling axis to increase BC aggressiveness.

## Introduction

Breast cancer (BC) is one of the most common malignancies in women worldwide. Despite the improvements in diagnosis and treatment strategies, BC remains the leading cause of cancer-related deaths in women [1]. Several explanations account for the high mortality rates of BC, of which distant metastasis is the principal cause [2]. The advent of novel genomic and transcriptomic analyses as well as single-cell gene expression analysis and animal modeling have yielded an unprecedented change in our understanding of BC metastasis. However, the molecular mechanisms underlying BC metastasis are still not entirely clear [2]. Therefore, systematic and in-depth research on the molecular mechanisms regulating BC metastasis would contribute to the identification of more effective metastasis-targeting agents.

In our previous investigation using gene expression microarray (GSE72307) [3], we showed that PRSS22 is upregulated in gastric cancer (GC) tissues with lymph node metastasis (LNM) compared with GC tissues without LNM. Furthermore, PRSS22 is initially confirmed as one novel oncogene promoting GC metastasis. PRSS22 (also known as Tryptase ε, BSSP-4) is a member of human serine proteases [4]. The serine proteases play vital functions in cancer development and progression. Previous study has demonstrated that PRSS22 readily converts zymogen pro-uPA (pro-urokinase-type plasminogen activator) into its mature and enzymatically active protease (uPA) [5]. In turn, PRSS22 improves smooth muscle cell migration by cleaving pro-uPA [5]. PRSS22 has also been shown to promote human hepatoma cancer [6]. However, the expression of PRSS22 in BC has not been investigated to date. In addition, the role of PRSS22 in BC tumorigenesis or progression and its underlying mechanism remain a big area to be explored.

## Methods and materials

### Tissue samples

Breast tissues, including 183 paraffin-embedded tissues and 52 fresh tissues, were collected from patients undergoing mammectomy at the Qilu Hospital of Shandong University (Jinan, Shandong, China), from 2005 to 2010. Total RNA was extracted from fresh breast tissues. The study was approved by the Research Ethics Committee of Shandong University of Medicine and is in accordance with the ethical guidelines of the World Medical Association Declaration of Helsinki. Informed consent was written by all patients prior to this study.

### Real-time quantitative polymerase chain reaction (RT-qPCR)

RNA samples were reverse transcribed to cDNA using reverse transcriptase cDNA synthesis kit (Toyobo, Osaka, Japan). We detected the expression levels of PRSS22 messenger RNA (mRNA) by qPCR using SYBR Green Real-time PCR Master Mix (Roche, Mannheim, Germany) on Bio-Rad CFX™ 96 C1000 Real-Time system. The relative expression of target gene was normalized to GAPDH expression, which yielded the 2^−ΔCt^ value.

### Immunohistochemistry (IHC)

The biotin–streptavidin horseradish peroxidase detection systems (SP-9000 Kits, ZSGB-BIO Technology Co. Ltd) were used to determine the expression of PRSS22 in BC tissues and breast normal tissues. The paraffin-embedded samples (4 μm) were incubated with PRSS22 antibody (1:300 dilution, Abcam, ab197158). The staining intensity and corresponding percentage were evaluated by two professional pathologists independently. The staining intensity was classified as follows: 0 (negative, no coloring), 1 (weak, light brown), 2 (moderate, brown), and 3 (strong, dark brown). Staining scores were then calculated as follows: score (maximum of 300) = (0 × percentage of no coloring cells) + (1 × percentage of weakly staining cells) + (2 × percentage of moderately staining cells) + (3 × percentage of strongly staining cells) [7]. Median score was selected as cut-off value to separate low expression group and high expression group.

### Cell culture and transfection

Human BC cell lines MDA-MB-231, MDA-MB-468, MDA-MB-453, MCF-7 and T47D were purchased from the American Type Culture Collection. BC cell lines were cultured in Leibovitz’s L15 medium (MDA-MB-231, MDA-MB-468 and MDA-MB-453) or Dulbecco’s modified Eagle’s medium (MCF-7 and T47D), supplemented with 10% fetal bovine serum (FBS; Gibco BRL, Grand Island, NY, U.S.). MDA-MB-231 and MDA-MB-468 cells were transfected with PRSS22 siRNA (RiboBio, Guangzhou, China) or the respective negative controls (NC), using X-tremeGENE transfection reagent (Roche, Indianapolis, IN, U.S.). The sequence of siPRSS22 was shown in Supplementary Table 1. pcDNA3.1-PRSS22 or the empty plasmid pcDNA3.1 (NC) were transfected with Lipofectamine 2000/3000 Transfection Reagent (Invitrogen, USA) according to the manufacturer’s instructions.

To prepare the conditional medium, cells were transiently transfected with PRSS22 plasmid separately. After 48 h, the culture medium was changed to serum-free medium. 24 h later, the conditional medium was collected and concentrated using ultrafiltration centrifuge tubes (MERCK MILLIPORE.UFC901024, 10 KD). The same amount of protein in concentrated conditional medium was used in following western blot experiments.

### Cell migration, invasion, proliferation, and apoptosis assays

Cell migration, invasion, proliferation (MTS and EdU) and apoptosis assays were performed as previously described [3, 8].

### Identification of PRSS22 proximal promoter region and transcriptional factors

The 2000-bp transcription start site (TSS) upstream sequence of PRSS22 was extracted from the UCSC Genome Browser(http://genome.ucsc.edu/). The putative PRSS22 promoter regions (−2000/0, −1012/0, −912/0, −562/0, −234/0) were PCR amplified from the genomic DNA of MDA-MB-231 cells, which were then inserted into the HindIII-NheI sites upstream of the firefly luciferase in the pGL3-Basic vector (Promega, USA). The constructs were named based on the location of the promoter fragments relative to the TSS: pGL3-2000 (P1), pGL3-1012 (P2), pGL3-912 (P3), pGL3-562 (P4) and pGL3-234 (P5). The luciferase activities were detected with the Dual luciferase Reporter system (Promega, USA) [9]. Then, we submitted the proximal core promoter sequence of PRSS22 to PROMO online program (http://alggen.lsi.upc.es/cgi-bin/promo_v3/promo/promoinit.cgi?dirDB=TF_6.4) and JASPAR program (http://jaspar.genereg.net/) to identify possible transcriptional factors. C/EBPβ, E2F1, ETS1 and MEIS1 have bene generated in our previous studies [10, 11].

### Chromatin immunoprecipitation (ChIP) assay

Chromatin immunoprecipitation (ChIP) assay was performed as previously described [9]. The precipitated DNA by anti-E2F1 antibody (Abcam, ab245308) or IgG antibody was quantified using qPCR. The primers were shown in Supplementary Table 2.

### Western blot

Western blot assay was conducted as previously described [12]. Primary antibodies were used for western blot in this study including PRSS22 (1:1000, Abcam, ab197158), E2F1 (1:1000, Abcam, ab245308), ANXA1 (1:1000, Abcam, ab214486), Flag (1:800, Cell Signaling Technology, 14793), p-ERK1/2 (1:500, Santa Cruz Biotechnology, sc-7383), ERK1/2 (1:500, Santa Cruz Biotechnology, sc-514302), p-AKT (1:500, Santa Cruz Biotechnology, sc-101629), p-p38 (1:500, Santa Cruz Biotechnology, sc-166182) and β-actin (1:2000, Santa Cruz Biotechnology, sc-8432).

### Vector construction and mimetic peptide synthetization

The PRSS22 wild-type plasmid pcDNA3.1-PRSS22 was generously provided by Dr. Liulei (Shandong University). ANXA1 plasmid was purchased from Vigene Biosciences. N-terminal deletion mutant ANXA1 was purchased from General Biosystems (Anhui) Co. Ltd. According to the literature [5], we designed PRSS22 protease-dead mutation plasmids (R49A and C139A/D141E) by quick change assay. The primers were shown in Supplementary Table 2. Flag tag (DYKDDDDK) was added to all PRSS22 wild-type and mutation plasmids at the C terminus. ANXA1 N-terminal-derived mimetic peptide (Ac2-26) was purchased from GL Biochem (Shanghai) Ltd.

### Detection of mimetic peptide function

BC cells were seeded in triplicate at a density of 5 × 10^5^ cells in 6-well plates. To synchronize the cell cycle, the culture medium was replaced with serum-free medium after cell adhesion. After 24 h, the culture medium was replaced with medium fortified with: (1) 10% FBS and Annexin A1 N-Terminal Derived Peptide Ac2-26 (Ac-AMVSEFLKQAWFIENEEQEYVQTVK, 1 μM, 5 μM, 10 μM) (GL Biochem) diluted in DMSO and (2) 10% FBS and same amount of DMSO. The concentration of Ac2-26 peptide was selected based on preliminary experiments and as reported elsewhere [13].

### In vivo mouse xenograft model

Six-week-old female BALB/c nude mice (Weitong Lihua Biotechnology, China) were randomly divided into two groups (n = 5 per group) for xenograft studies. We transfected MDA-MB-231 cells with the lentiviral PRSS22 knockdown vector (LV-sh-PRSS22) or control vector (LV-NC) (Shanghai Genechem Co., Ltd., China). A total of 1×10^7^ lentivirus transfected MDA-MB-231 cells were injected into the mammary fat pads of mice. The tumor volumes were measured as V = (Tumor length × Width^2^)/2 every 5 days after inoculation. Eight weeks after injection, the mice were sacrificed, and tumor nodules were isolated for further analysis. All protocols were approved by the Committee on the Ethics of Animal Experiments of Basic Medical Sciences, Shandong University.

### Co-immunoprecipitation (Co-IP) assay

MDA-MB-231 cells were transfected with pcDNA3.1-PRSS22 wild-type and protease-dead mutation plasmids (R49A, C139A/D141E). After 48 h, 1000 μg of total proteins were incubated with Flag Tag Immunomagnetic Beads (Sino Biological, TB101274) at 4 °C overnight. The beads were then washed thrice with 300 μL 5 × TBST buffer and once with 300 μL of ddH_2_O. The binding proteins were subjected to SDS-PAGE gel and then the gel was silver stained to detect differentially expressed bands. The bands were analyzed by mass spectrometry.

### Data analysis

GraphPad Prism 8.0 (GraphPad Software, USA) was used for data analyses. Pearson’s chi-squared (χ2) test or Fisher’s exact test was used to determine the correlation between PRSS22 expression and the clinicopathological parameters. The Student’s *t* test and one-way ANOVA were used to analyze the differences between two groups and multiple groups, respectively. Each experiment was repeated three times independently. *P* < 0.05 was considered statistically significant.

## Results

### PRSS22 is upregulated in human BC tissues and is associated with metastasis

**Fig. 1:**
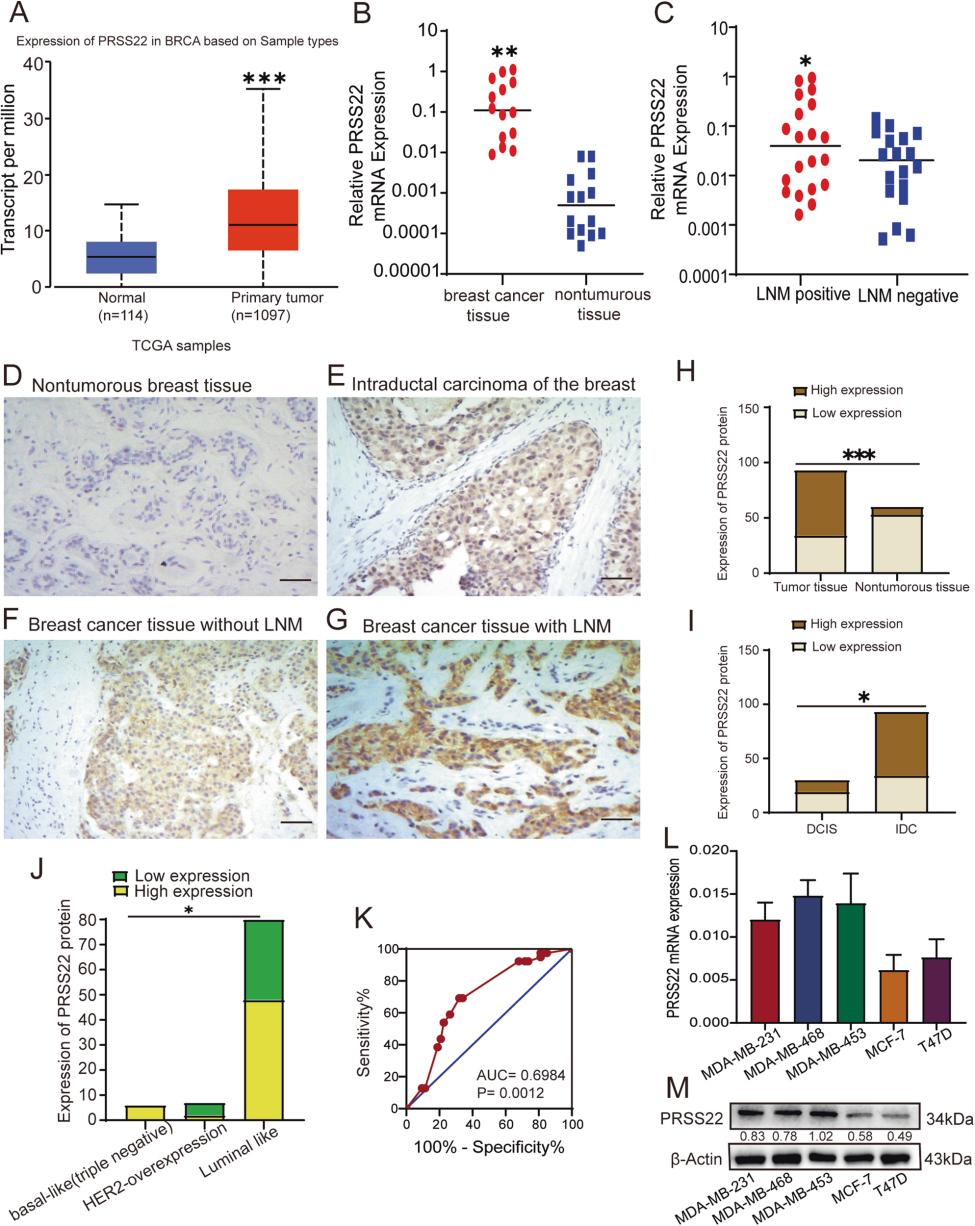
Identification and validation of PRSS22 as a tumor-associated gene in BC. **A** PRSS22 expression is markedly elevated in human BC samples compared with normal tissues from the TCGA databases using UALCAN (BRCA: Breast invasive carcinoma, Comparison: Normal-vs-Primary tumor, Statistical significance:1.6244783296E-12). **B** The expression of PRSS22 in tumor tissues (*n* = 14) and paired non-tumor tissues (*n* = 14) detected by RT-qPCR (*t* test, *P* = 0.0056). **C** The expression of PRSS22 in tumor tissues with lymph node metastasis (LNM) (*n* = 20) and without LNM (*n* = 18) detected by RT-qPCR (*t* test, *P* = 0.019). The median expression of each group was indicated by horizontal lines. **D**–**G** The expression of PRSS22 was confirmed immunohistochemically in BC paraffin-embedded samples. PRSS22-positive staining was predominantly localized in the cytoplasm and membrane. PRSS22 is negative in normal gastric mucosa, weak intensity with low positivity rate in primary BC tissues without LNM, strong intensity with high positivity rate in primary BC tissues with LNM (magnification ×200, Scale bars, 50 μm). **H** Immunohistochemistry showed that PRSS22 expression is significantly upregulated in BC tissues compared with nontumorous breast tissues (*P* < 0.0001). **I** Compared with ductal carcinoma in situ (DCIS), the expression of PRSS22 in invasive ductal carcinoma (IDC) of BC tissues increased significantly (*P* = 0.0118). **J** Immunohistochemistry showed that PRSS22 expression is significantly upregulated in molecular classification of triple-negative BC (*P* < 0.05). **K** Area under the receiver operator characteristic (ROC) curve for PRSS22 in differential diagnosis of BC with and without LNM (AUC = 0.6984, *P* = 0.0012). **L, M** The expression of PRSS22 in triple-negative metastatic cell lines (MDA-MB-231, MDA-MB-468 and MDA-MB-453) and luminal cell lines (T47D and MCF-7) were performed by RT-qPCR (**L**) and Western blot (**M**). *P < 0.05, **P < 0.01, ***P < 0.001, ns not significant.

The UALCAN online software (http://ualcan.path.uab.edu/analysis.html) was used to analyze published data on PRSS22 expression in BC from TCGA. The results reveal that the expression of PRSS22 is markedly elevated in human BC samples compared with breast normal tissues (Fig. 1A, *n* = 1097 and *n* = 114, *P* < 0.05). In our study, we examined PRSS22 mRNA expression in fresh BC tissues and nontumorous tissues, the results indicated that PRSS22 was not only significantly upregulated in BC tissues compared with breast normal tissues samples (Fig. 1B), but also expressed at higher levels in patients with LNM (Fig. 1C).

By IHC assay, PRSS22 was located mainly in cytoplasm and cell membrane (Fig. 1D–G). Similar to the results of PRSS22 mRNA analysis, PRSS22 protein expression was downregulated in nontumorous breast tissues (Fig. 1H), and upregulated in BC tissues, particularly in BC with LNM (Table 1). Compared with ductal carcinoma in situ (DCIS), PRSS22 expression significantly increased in invasive ductal carcinoma (IDC) of the breast (Fig. 1I). These results indicated that PRSS22 expression was up-regulated gradually from nontumorous breast tissues to BC tissues without LNM to BC tissues with LNM (Table 1 and Fig. 1D–G). Correlation analysis showed that upregulated PRSS22 expression was strongly correlated with molecular classification (Fig. 1J), positive LNM (*P* = 0.0021), clinical stage (*P* = 0.0080), and histological grading (*P* = 0.0133), whereas there was no correlation between PRSS22 expression and other characteristics such as age, tumor size, as well as the expression of ER, PR, and HER2 (Table 1). Receiver operator characteristic (ROC) curve showed that PRSS22 expression could be used to distinguish BC patients with LNM from BC patients without LNM, with an area under the ROC curve (AUC) of 0.6984 (*P* = 0.0012) (Fig. 1K).

**Table 1:** Relationship between PRSS22 protein expression and clinicopathological parameters.

Variables	Total	PRSS22 status		*P* value
		Low	High	
Age				
≤35	1	1	0	0.3319
36-55	60	23	37	
>55	32	10	22	
Diameter (cm)				
≤2.5	51	23	28	0.0834
>2.5	42	11	31	
Lymph node metastases				
Negative	40	22	18	**0.0021**
Positive	53	12	41	
pTNM stage				
I + II	57	27	30	**0.0080**
III + IV	36	7	29	
ER				
Negative	18	7	11	å 0.9999
Positive	75	27	48	
PR				
Negative	19	8	11	0.6012
Positive	74	26	48	
HER2				
Negative	79	26	53	0.1510
2+	10	5	5	
3+	4	3	1	
Histological grading				
I	14	9	5	**0.0133**
II	63	23	40	
III	16	2	14	

Bold values indicate statistical significance *P* < 0.05.

Furthermore, we observed significantly higher PRSS22 expression level in triple-negative metastatic cell lines MDA-MB-231, MDA-MB-468 and MDA-MB-453 than luminal cell lines T47D and MCF-7 (Fig. 1L, M). This observation suggested that the high expression of PRSS22 is associated with increased BC aggressiveness. To sum up, these findings demonstrated that PRSS22 may play an important role in the pathogenesis of BC.

### E2F1 activates PRSS22 transcription in BC cells

**Fig. 2:**
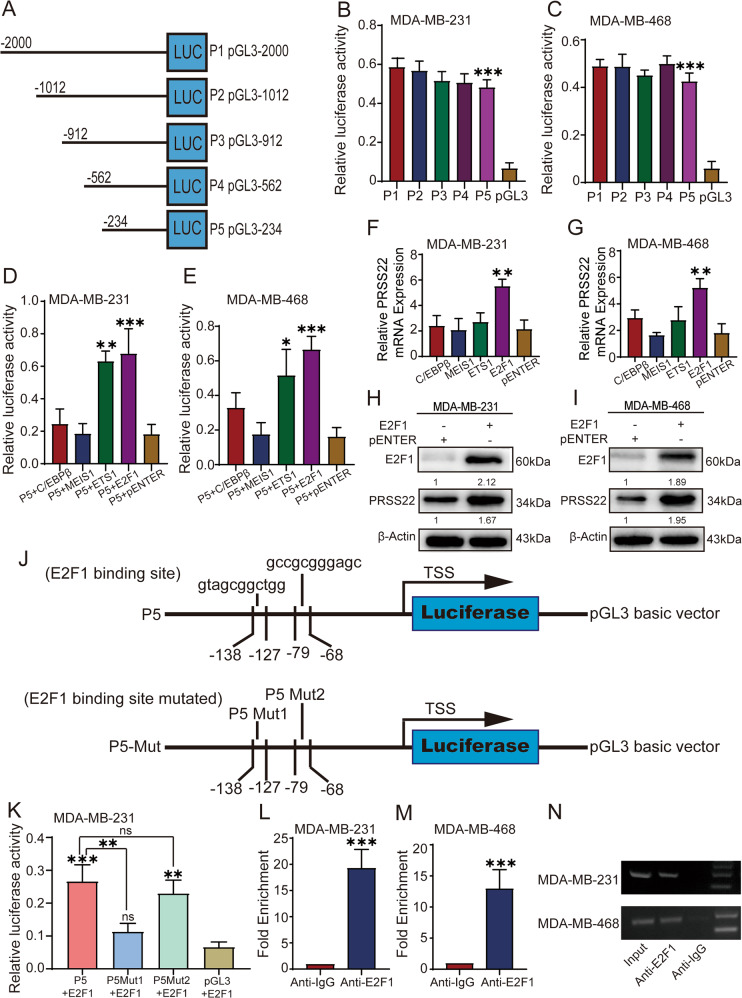
E2F1-activated PRSS22 transcription in BC cells. **A** Schematic diagram of luciferase vectors pGL3-2,000(P1), pGL3-1,012(P2), pGL3-912(P3), pGL3-562(P4) and pGL3-234(P5). **B**, **C** Transcriptional activity analysis of the PRSS22 promoter fragments in BC cells revealed that the upstream 234 bp(P5) is core promoter region for PRSS22 transcription. **D**, **E** Luciferase activity assay demonstrated that E2F1 and ETS1 increased promoter activities of P5 in BC cells. **F**–**I** The influence of E2F1 on PRSS22 were measured by RT-qPCR (**F**, **G**) and Western blot (**H**, **I**). **J** Two primers that covered the E2F1 binding sites at the regions −127 to −138 bp, −68 to −79 bp were designed (up). Schematic diagram of the luciferase reporter construct containing the human PRSS22 promoter and the mutant construct (P5 Mut1, P5 Mut2) containing the basal promoter in which the presumed binding sites were mutated (bottom). **K** The relative luciferase activity of the PRSS22 promoter was decreased when the predicted E2F1-binding site (P (−127 to −138) Luc-E2F1-1) was mutated (P5 Mut1). **L**–**N** ChIP-qPCR results indicated that the promoter amplicons in the E2F1-binding site precipitated by anti-E2F1 antibody were more enriched than those precipitated by anti-IgG antibody. Data are shown as mean ± SD of three independent experiments, *P < 0.05, **P < 0.01, ***P < 0.001, ns not significant.

To elucidate the mechanism of PRSS22 overexpression in BC, we performed promoter analysis to search potential transcription factor of PRSS22. The promoter activities of the region ~2000 bp upstream of its transcription start site (TSS) and five deletion constructs [named P1 (pGL3-2,000), P2 (pGL3-1,012), P3 (pGL3-912), P4 (pGL3-562) and P5 (pGL3-234)] were compared with the luciferase reporter vector pGL3-Basic in BC cells using dual-luciferase reporter assays (Fig. 2A). No statistically significant difference in luciferase activity was observed between pGL3-2000 and the deletion constructs, including pGL3-1,012, pGL3-912, pGL3-562, and pGL3-234. A significant decrease (90%) in luciferase activity from pGL3-234 to pGL3-Basic indicated that the upstream 234 bp region may be the basal promoter of PRSS22 (Fig. 2B, C).

PROMO and JASPAR online software predicted that E2F1, MEIS1, ETS1, and C/EBPβ, which had high scores, were putative transcription factors (TFs) that bind to the promoter region encompassing positions −234 to 0. Overexpression of E2F1 and ETS1 markedly improved the promoter activities of P5 (Fig. 2D, E). However, RT-qPCR showed that only E2F1 significantly enhanced PRSS22 mRNA expression in BC cells (Fig. 2F, G). Western blot analysis revealed that E2F1 promoted PRSS22 protein expression (Fig. 2H, I). To explore direct binding interaction between E2F1 and the PRSS22 promoter, we constructed two binding site deletion mutations, namely, P (−127 to −138) Luc-E2F1-1 designated as P5 Mut1 and P (−68 to −79) Luc-E2F1-2 designated as P5 Mut2 (Fig. 2J). The primers are shown in Supplementary Table 2. Dual-luciferase reporter assays showed that the P5 Mut1 mutation vectors led to an approximate 60% decrease in promoter activity, indicating that the E2F1-binding p (−127 to −138) Luc-E2F1-1 site is responsible for PRSS22 transcription (Fig. 2K). Moreover, chromatin immunoprecipitation (ChIP)-PCR assays indicated that E2F1 directly binds to the PRSS22 promoter region (Fig. 2L–N).

### PRSS22 promotes BC cells invasion and metastasis in vitro and in vivo

**Fig. 3:**
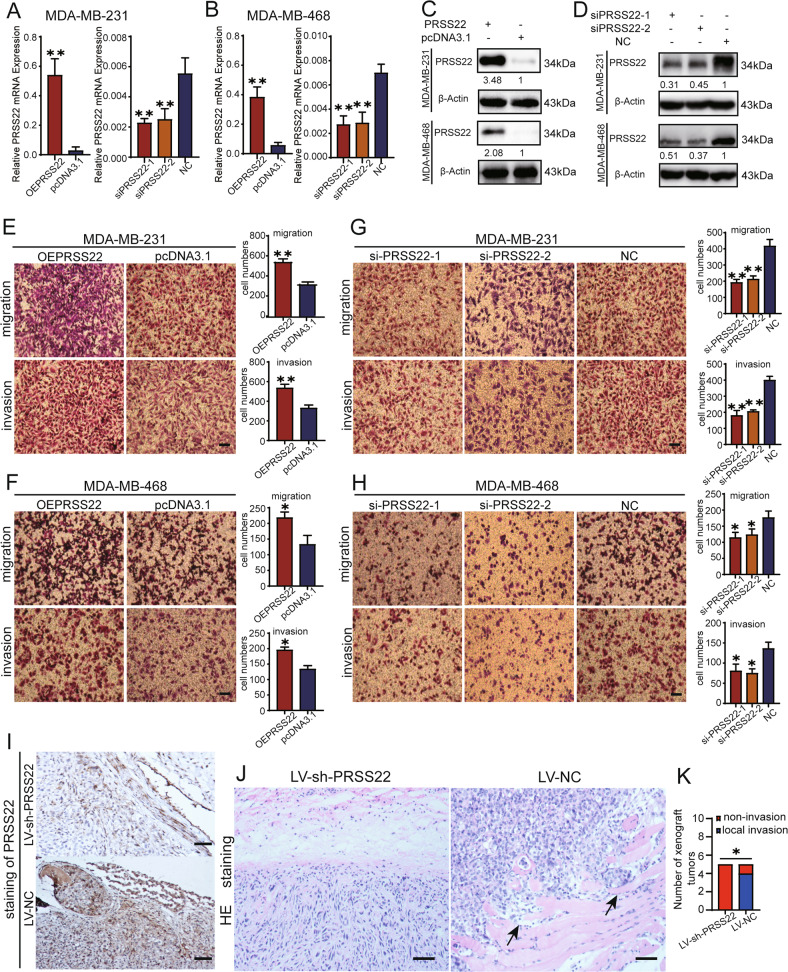
PRSS22 promotes BC cells migration and invasion. **A**–**D** The overexpression efficiency and knockdown efficiency of PRSS22 in BC cells. **E**–**H** Transwell assays showed that overexpression of PRSS22 enhanced the migratory and invasive ability of BC cells, while knockdown of PRSS22 inhibited migratory and invasive capacity (Scale bars, 100 μm). Each experiment was performed in triplicate. **I** Immunohistochemistry staining of PRSS22 in xenograft tumors (magnification ×200, Scale bars, 50 μm). **J** The xenograft tumors of LV-NC group displayed local invasion, with cancer cells invading into the surrounding muscle tissues (the black arrow), the tumors of LV-sh-PRSS22 group were well-encapsulated (HE magnification ×200, Scale bars, 50 μm). **K** The quantification of local invasion of xenograft tumors and associated statistical analyses. Data are shown as mean ± SD of three independent experiments, *P < 0.05, **P < 0.01, ***P < 0.001, ns not significant.

To study the roles of PRSS22 in BC, we overexpressed and knocked down PRSS22 expression separately in MDA-MB-231 and MDA-MB-468 cells. First, we detected the overexpression and knockdown efficiency of PRSS22 expression (Fig. 3A–D). Migration and invasion assays showed that the overexpression of PRSS22 enhanced migration and invasion compared with the control group, whereas PRSS22 knockdown markedly suppressed the migratory and invasive abilities of BC cells (Fig. 3E–H).

To explore the effect of PRSS22 on cell invasion in vivo, we injected MDA-MB-231 cells transfected with LV-sh-PRSS22 or LV-NC subcutaneously into mice. Immunohistochemistry (IHC) analysis confirmed that the expression of PRSS22 was knocked down in LV-sh-PRSS22 group xenograft tumors (Fig. 3I). Histological examination confirmed that tumors in the LV-NC group displayed local invasion, with islands of cancer cells invading muscle tissues. However, those in the LV-sh-PRSS22 group were well-encapsulated or exhibited non-invasion (Fig. 3J, K). These results indicate that knocking down PRSS22 could inhibit BC progression by reducing their invasion capability.

### PRSS22 has no effect on BC cells proliferation or apoptosis

Additionally, we examined potential effects of PRSS22 on BC cell proliferation and apoptosis. MTS, EdU, and Annexin V-FITC assays indicated that overexpression or suppression of PRSS22 in BC cells does not affect BC cell growth and apoptosis (Supplementary Fig. 1A–H). In line with these results, subcutaneous implantation mouse models also showed no significant differences in the mass of primary tumors between the LV-sh-PRSS22 and LV-NC groups (Supplementary Fig. 1J–L). These results revealed that PRSS22 does not affect BC tumor growth.

### PRSS22 promotes cancer progression via its protease activity

**Fig. 4:**
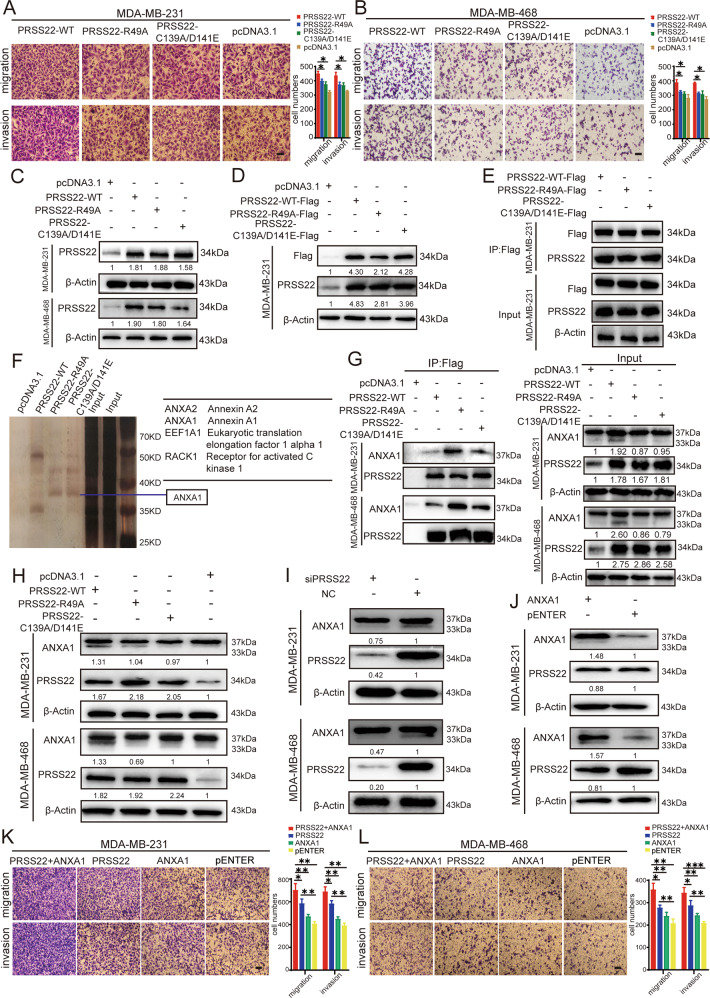
PRSS22 binds to ANXA1 protein and promotes the cleavage of ANXA1 in BC cells. **A**, **B** Transwell assays showed that PRSS22 protease-dead mutation plasmids lost their function in promoting migration and invasion (Scale bars, 100 μm). **C** Western blot assays were used to identify the overexpression efficiency of PRSS22-WT, PRSS22-R49A and PRSS22-C139A/D141E in BC cells. **D** Western blot assays showed that the overexpression efficiency of Flag tag (DYKDDDDK). **E** Western blot assays were used to verify that DYKDDDDK (Flag®) Tag Immunomagnetic Beads are able to precipitate PRSS22 protein. **F** Silver staining of the PRSS22-protein complex by incubation of PRSS22 with protein extracts from MDA-MB-231 cell. The line shows the differential band that was present when the protein extracts were incubated with PRSS22-WT compared with PRSS22-R49A and PRSS22-C139A/D141E. **G** Co-IP and Western blot assays were used to verify that PRSS22 specifically binds to ANXA1 in BC cells. **H**, **I** Western blot assay was performed to confirm that PRSS22-WT could promote the cleavage of ANXA1, whereas PRSS22-R49A or PRSS22-C139A/D141E have no obvious effect on the cleavage of ANXA1. The data of cleaved ANXA1 proteins are shown as mean ± SD of three independent experiments. **J** Western blot assays showed that ANXA1 has no obvious effect on the expression level of PRSS22 protein. **K**, **L** Transwell assays showed that BC cells were co-transfected with PRSS22 and ANXA1 have stronger migration and invasion ability than those transfected with only PRSS22 or ANXA1 (Scale bars, 100 μm). Data are shown as mean ± SD of three independent experiments, *P < 0.05, **P < 0.01, ***P < 0.001, ns not significant.

PRSS22 is a serine protease that can catalyze substrate proteins. Enzyme activity is significant for PRSS22 to serve its important biological function. A previous study indicated that Arg-49, Cys-139, and Asp-141 are key amino acid sites that are responsible for its enzyme activity. Arg-49 is in the propeptide domain and controls PRSS22 self-activation. Cys-139 and Asp-141 are in the active center of PRSS22. The R49A mutant inhibits PRSS22 autoactivation and loses its enzyme activity. And the C139A/D141E mutant could hardly catalyze substrates [5]. To explore whether PRSS22 promotes BC invasion and migration mainly through its enzymatic activity, we constructed PRSS22 protease-dead mutation plasmids (R49A and C139A/D141E). MDA-MB-231 and MDA-MB-468 cells were separately transfected with the PRSS22 wild-type and protease-dead mutation plasmids. Transwell assays demonstrated that PRSS22 protease-dead mutation plasmids lost their function in promoting BC cells migration and invasion (Fig. 4A, B). These results indicated that PRSS22 mainly promoted cancer progression through its enzyme activity.

### ANXA1 is the substrate of PRSS22 in BC cells

Functional experiments showed that PRSS22 promotes BC migration and invasion via its enzymatic activity. Therefore, we attempted to identify the candidate substrate of PRSS22 in BC and explored the underlying mechanism. Co-IP assay together with SDS-PAGE gel silver staining were used to explore the proteins that may directly interact with PRSS22 in MDA-MB-231 cells, and different bands were analyzed by protein mass spectrometry (Fig. 4E, F).

Protein mass spectrometry analysis identified a total of 125 proteins (45 proteins in the PRSS22 group, 46 proteins in the R49A group, and 34 proteins in the C139A/D141E group). Keratin and housekeeping proteins were excluded from further analysis. According to literature reports and mass spectrometry scores, we evaluated two putative binding proteins, ANXA1 (Annexin A1) and ANXA2 (Annexin A2). CO-IP and western blot assays demonstrated that PRSS22 specifically binds to ANXA1 (Fig. 4G).

ANXA1, a 37-kDa protein, is reported to be cleaved by protease 3 at the N-terminal region and generates a truncated 33-kDa ANXA1 [14]. To explore whether the interaction between PRSS22 and ANXA1 promotes the cleavage of ANXA1, we designed an intracellular protein cleavage experiment. Western blot confirmed that PRSS22 could promote the cleavage of ANXA1 (showing cleaved ANXA1 proteins, 33 kDa), whereas protease-dead mutations R49A and C139A/D141E have no obvious effect on the cleavage of ANXA1 (Fig. 4H, I). The above results further confirmed that PRSS22 promotes the cleavage of ANXA1 mainly through its enzymatic activity. To determine whether ANXA1 regulates PRSS22 expression, we transiently transfected ANXA1 in BC cells to assess changes in PRSS22 expression. The results demonstrated that ANXA1 had no obvious effect on PRSS22 expression (Fig. 4J). In addition, transwell assays showed that BC cells co-transfected with PRSS22 and ANXA1 exhibited stronger migration and invasion ability than those transfected with only PRSS22 or ANXA1 (Fig. 4K, L). These findings suggest that ANXA1 is the downstream protein of PRSS22 and promotes BC aggressiveness.

### PRSS22 combined with ANXA1 regulates BC cells invasion via FPR2/ERK axis

**Fig. 5:**
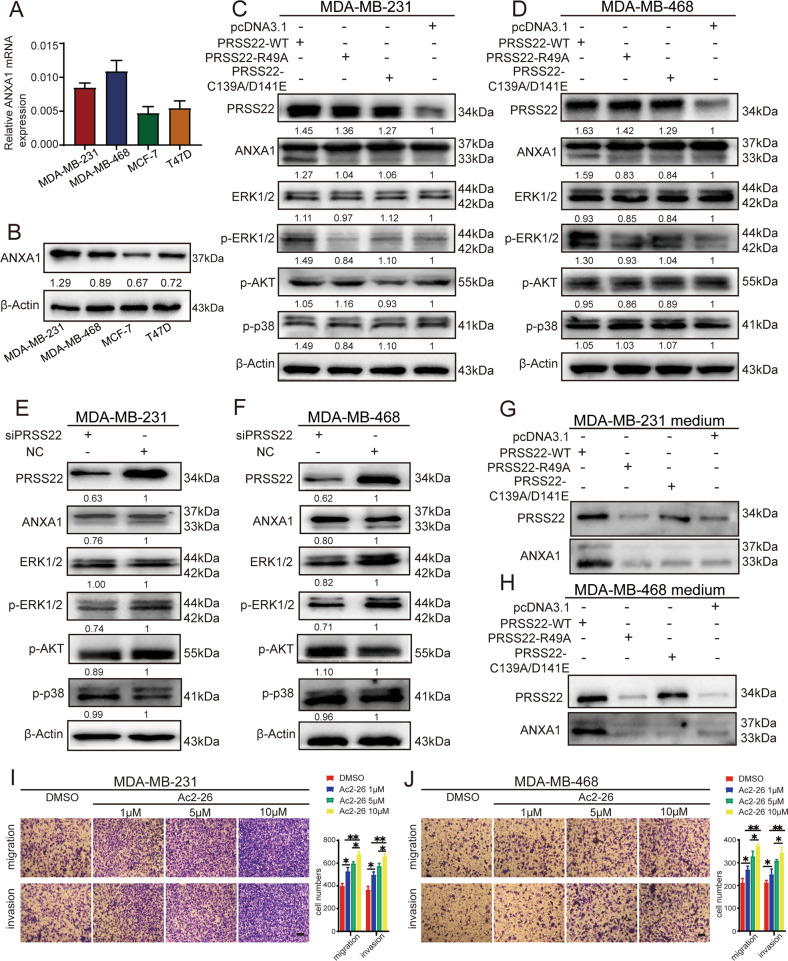
PRSS22 combined with ANXA1 regulates BC cells migration and invasion by ERK 1/2 signaling pathway. **A**, **B** The expression of ANXA1 in triple-negative metastatic cell lines (MDA-MB-231, MDA-MB-468 and MDA-MB-453) and luminal cell lines (T47D and MCF-7) were performed by RT-qPCR (**A**) and Western blot (**B**). **C**, **D** Western blot analysis of the different signaling modules downstream of PRSS22 in BC cells expressing PRSS22-WT, PRSS22-R49A, PRSS22-C139A/D141E or control cells. **E**, **F** Western blot analysis of the different signaling modules downstream of PRSS22 in BC cells with or without PRSS22 knockdown. **G**, **H** The expression of PRSS22 and ANXA1 in concentrated conditioned medium of BC cells expressing PRSS22-WT, PRSS22-R49A, PRSS22-C139A/D141E or control were performed by Western blot. **I**, **J** Transwell assays confirmed that Ac2-26 induced a dose-dependent increase in migration and invasion (Scale bars, 100 μm). Data are shown as mean ± SD of three independent experiments, *P < 0.05, **P < 0.01.

Next, we determined the expression of ANXA1 in BC cell lines. Consistent with the expression of PRSS22, ANXA1 expression was relatively higher in triple-negative metastatic cell lines MDA-MB-231 and MDA-MB-468 compared with the luminal cell lines MCF-7 and T47D (Fig. 5A, B). It was recognized that ANXA1 could be cleaved by protease 3 at the N-terminal region and generate a truncated 33-kDa ANXA1. We found that PRSS22 increased the levels of an ANXA1-immunoreactive band at 33 kDa in cell lysates (Fig. 5C–F) and concentrated conditioned medium (Fig. 5G, H). Interestingly, the expression of 33-kDa ANXA1 in the conditioned medium is very high. These results suggested that cleavage products (33-kDa ANXA1) of ANXA1 were secreted out of the cells.

ANXA1 is susceptible to proteolytic cleavage. Previously research showed that human leukocyte elastase cleaved ANXA1 into two parts, a truncated 33-kDa ANXA1 and an N-terminal peptide [15]. The N-terminal peptide played major roles in receptor activation and desensitization [16, 17]. To explore whether the 33-kDa ANXA1 plays vital functions in BC, MDA-MB-231 and MDA-MB-468 cells were transiently transfected with 33-kDa truncated ANXA1. However, the results showed that the 33-kDa ANXA1 did not alter cell migration and invasion (Supplementary Fig. 2A, B). Therefore, we hypothesized that the N-terminal peptide, another product of ANXA1 cleavage, might play promoting effect in BC. As expected, transwell assays showed that ANXA1 N-terminal-derived mimetic peptide (Ac2-26) could promote migration and invasion ability of BC cells. In addition, Ac2-26 induced a dose-dependent increase in migration and invasion (Fig. 5I, J).

Ac2-26 has been shown to regulate leukocytes and other tumor cell migratory events through interactions with formyl peptide receptors (FPRs), and thus we examined the expression of FPR1, FPR2, and FPR3 in BC cells by RT-qPCR. The results showed that the expression of FPR1 and FPR2 is higher than FPR3 in BC cells (Supplementary Fig. 2C). In addition, to explore whether the cleavage products of PRSS22 play important roles in promoting BC mainly through FPRs, cells were co-transfected with PPSS22 plasmid and control siRNA or siRNA against FPR1, FPR2, or FPR3 (Supplementary Fig. 2D, E). Transwell assays showed that migration and invasion ability were reduced by co-transfection of PRSS22 and FPR2 siRNA. In contrast, FPR1 or FPR3 knockdown did not alter cell migration and invasion (Supplementary Fig. 2F, G). These data indicate that FPR2 is the downstream molecule of PRSS22, that may mediate BC cell migration and invasion.

Previous studies indicated that the activation of FPRs may stimulate several signaling pathways, including ERK1/2 (extracellular signal-regulated kinase 1/2), PI3K (phosphoinositide 3-kinase)/AKT, and p38 MAPK [18, 19]. Here, we examined whether the ERK1/2, PI3K, or p38 MAPK pathway is implicated in PRSS22/ANXA1/FPR2-induced cell phenotypes. Our results showed marked upregulation of phosphate-ERK1/2 (p-ERK1/2) in PRSS22-overexpressing BC cells, whereas PRSS22 knockdown significantly decreased the expression of p-ERK1/2 (Fig. 5C–F). No significant changes in phosphorylated AKT and p38 expression were observed after PRSS22 overexpression or knockdown (Fig. 5C–F).

**Fig. 6:**
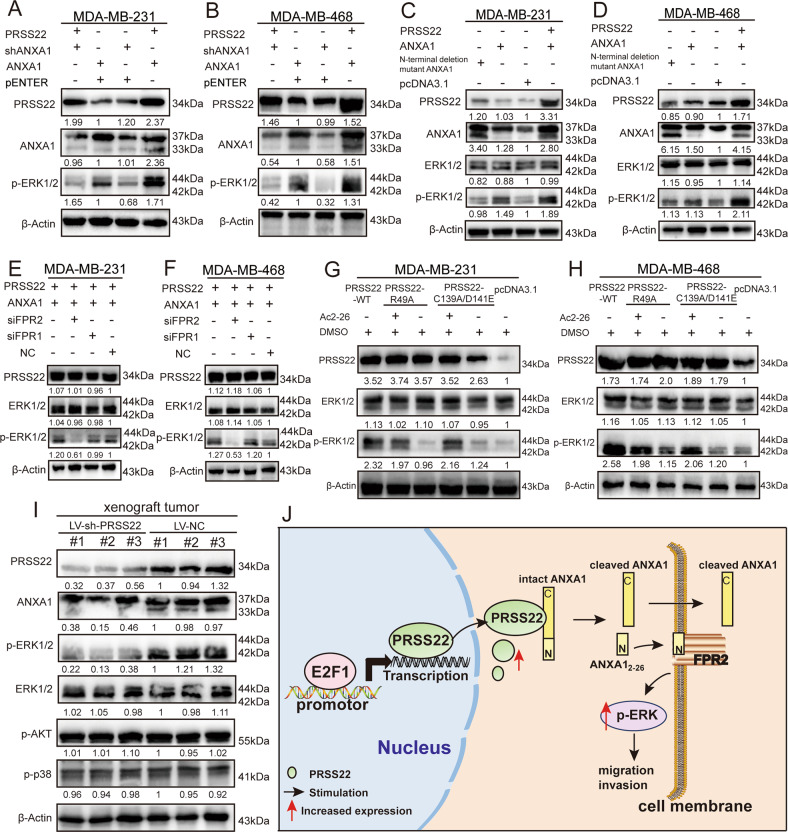
PRSS22 combined with ANXA1 regulates BC cells invasion via FPR2/ERK axis. **A**, **B** Western blot assays showed that shRNA of ANXA1 inhibited PRSS22-activated phosphorylation of ERK. **C**, **D** N-terminal deletion mutant ANXA1(33 kDa ANXA1) upregulation did not promote p-ERK1/2 expression. **E**, **F** Western blot showed that FPR2 knockdown reversed the high expression of p-ERK1/2 induced by PRSS22 and ANXA1 overexpression. **G**, **H** BC cells expressing PRSS22-R49A and PRSS22-C139A/D141E were incubated with the peptide AC2-26 and p-ERK1/2 expression was analyzed by Western blot. **I** The expression of PRSS22, ANXA1, p-ERK1/2, ERK1/2, p-AKT and p-p38 protein in xenograft tumors were detected by Western blot. **J** Working model of the upstream regulatory and function mechanisms of PRSS22 in BC progression.

Furthermore, we investigated whether p-ERK1/2 upregulation mediated by PRSS22 is exerted via ANXA1-FPR2. Western blot assays showed that ANXA1 knockdown led to p-ERK1/2 downregulated expression (Fig. 6A, B). However, the upregulation of N-terminal deletion mutant ANXA1 (33-kDa ANXA1) did not promote the expression of p-ERK1/2 (Fig. 6C, D). BC cells expressing PRSS22-R49A and PRSS22-C139A/D141E were incubated with the peptide AC2-26. Figure 6G and H show that Ac2-26 increases p-ERK1/2 expression. FPR2 knockdown could reverse the upregulated expression of p-ERK1/2 that was induced by PRSS22 and ANXA1 overexpression (Fig. 6E, F).

To confirm whether PRSS22 exhibits its important biological functions via the ANXA1/FPR2/ERK-signaling pathway in vivo, we established subcutaneous xenograft models in female nude mice. Western blot confirmed that cleaved (33 kDa) ANXA1 protein expression levels decreased in LV-sh-PRSS22 tumors. Moreover, PRSS22 knockdown inhibited p-ERK1/2 expression in LV-sh-PRSS22 tumors without affecting the expression of p-AKT and p-p38 (Fig. 6I). Taken together, we demonstrated that PRSS22 cleaved ANXA1 at the N-terminal region and generated an N-terminal peptide. The combination of the N-terminal peptide and FPR2 initiated the subsequent ERK1/2 phosphorylation cascade that in turn increased BC aggressiveness.

## Discussion

Serine proteases play essential roles in various physiological processes, such as immunity, blood coagulation, nutrient digestion, fibrinolysis, cell migration, and reconstitution of extracellular matrices [20]. PRSS22 was initially identified as a member of the serine protease family [4]. Under physiologic conditions, human PRSS22 is expressed in the airways in a developmentally regulated manner [4]. In terms of tumors, PRSS22 has been known to be highly expressed in hepatocellular carcinoma and is related to metastasis and invasion [6]. In this study, we first determined that PRSS22 expression is upregulated in an increasing order in non-tumorous breast tissues, non-metastatic BC tissues, and metastatic BC tissues.

To explore the mechanism of PRSS22 upregulation in BC, we defined the proximal promoter of PRSS22, and confirmed that E2F1 is a transcription factor that upregulates PRSS22 in BC. E2F transcription factor 1 (E2F1), a member of the E2F family, has been initially described as an important regulator of apoptosis and proliferation in cells [21, 22]. Moreover, multiple studies have revealed the critical roles of E2F1 in cancer progression. E2F1 can increase the proliferation and metastasis of clear cell renal cell carcinoma (ccRCC) through SREBP1-induced aberrant lipid metabolism [23]. E2F1 also promotes the invasion of BC and is associated with poor prognosis in BC [24–26]. Here, our findings provide new insights into the E2F1 transcriptional regulation of PRSS22. Dual luciferase assays, bioinformatics analyses, and ChIP assays indicated that the E2F1 transcription factor directly binds to the PRSS22 promoter region (upstream 138 bp to 127 bp region) and activates its transcription.

Currently, the underlying molecular mechanisms of PRSS22 in BC progression remain unclear. We demonstrated that PRSS22 directly binds to ANXA1, which was confirmed by Co-IP assays. The combined effect of PRSS22 and ANXA1 promoted migration and invasion of BC cells. ANXA1 is a member of calcium/phospholipid-binding and actin regulatory proteins with diverse functions [27]. Previous studies have detected that intact (37 kDa) and cleaved (33 kDa) ANXA1 proteins are accompanied by elastase [28]. In the cytosol, ANXA1 mainly occurs as an N-terminal-intact form (37 kDa). It could be cleaved and released as an N-terminal-clipped form (33 kDa) [14]. More recent studies have shown that the ANXA1 N-terminal peptide, Ac2-26, promotes the migration of WS1 human skin fibroblasts [29] and the invasion of SKCO-15 colorectal adenocarcinoma cells through interaction with FPRs [30]. Our results indicate that ANXA1 cleaved forms (33 kDa) are upregulated in PRSS22 overexpressed cell lysates and conditional medium; however, PRSS22 had no obvious effect on the expression of ANXA1 N-terminal-intact form (37 kDa). These findings indicate that PRSS22 can promote the cleavage of ANXA1. Cells secreted the cleaved form of ANXA1 (33 kDa). Interestingly, Ac2-26 promoted MDA-MB-231 and MDA-MB-468 cell migration and invasion. Cleaved ANXA1 (33 kDa) did not alter cell migration and invasion.

In addition, the results of recent reports on the expression of ANXA1 in BC are discordant. Some studies have shown that ANXA1 expression is associated with a highly invasive BC subtype [31, 32]. Our results revealed that ANXA1 was relatively highly expressed in triple-negative metastatic cell lines MDA-MB-231 and MDA-MB-468 compared with the luminal cell lines MCF-7 and T47D. Therefore, we consider that ANXA1 may be involved in BC progression. Furthermore, we found that knocking down FPR2 blocked PRSS22-activated cell migration and invasion. These findings illustrated that ANXA1 was cleaved by PRSS22 in BC cells, and ANXA1 N-terminal-clipped 33-kDa form was released. ANXA1 N-terminal peptide bound to FPR2 promotes BC progression. ANXA1 N-terminal-clipped 33-kDa form did not generate any detectable alterations in BC function.

Furthermore, we assessed the expression of key proteins in the downstream signal pathways of FPR2. Our results showed that the expression of p-ERK1/2 was markedly upregulated in PRSS22-overexpressing BC cells. Previous studies have reported the role of p-ERK1/2 in tumor proliferation, invasion and metastasis, and the function of the MAPK/ERK signaling pathway in tumor extracellular matrix degradation and tumor angiogenesis [33–35]. With respect to BC, earlier studies have suggested an involvement of activated MAP kinases in carcinogenesis and progression. Phosphorylation of ERK1/2 mitogen-activated protein kinase is associated with poor response to anti-hormonal therapy and decreased patient survival in BC [36]. Previous studies have shown that the ERK signaling pathway is important for Activator protein-1 (AP-1) activation [37]. Several AP-1 proteins, including c-Jun, JunB, c-Fos and ATFs, were suggested to regulate cyclin D1 transcription [38–41]. The cyclin D1/CDK4/Rb pathway is recognized as a key pathway in the regulation of E2F1 activity, leading to the release of E2F1 [42]. E2F1 could in turn directly bind to the PRSS22 promoter region and further activate the transcription of PRSS22, thus forming a positive feedback loop that drives the malignant behavior of BC. In this study, shRNA of ANXA1 blocked PRSS22-activated ERK phosphorylation. In addition, FPR2 knockdown reversed the upregulated expression of p-ERK1/2 that was induced by PRSS22 and ANXA1 overexpression. Our results indicated that PRSS22 promotes BC migration and invasion via ANXA1/FPR2/ERK axis.

In summary, the present study shows that PRSS22 is upregulated in BC, particularly in cases with positive LNM or higher clinical stages. PRSS22 upregulation is associated with increased aggressiveness of BC. PRSS22, which is upregulated by the E2F1 transcription factor, could directly combine with ANXA1 to promote ANXA1 cleavage that subsequently initiates FPR2-ERK cascades to improve the aggressiveness of BC cells (Fig. 6J). These findings suggest that PRSS22 plays important roles in BC progression and thus could be a candidate biomarker for BC treatment.

## Supplementary information

Author Contribution Statement

Supplementary Figure legends

Supplementary Figure1

Supplementary Figure2

Supplementary Table 1

Supplementary Table 2

Original Data File

reproducibility checklist

## Data Availability

All datasets generated or analyzed during this study are included in this published article and its supplementary information files. Additional data are available from the corresponding author on reasonable request.
