# Supplementary material for: E2F1-initiated transcription of PRSS22 promotes breast cancer metastasis by cleaving ANXA1 and activating FPR2/ERK signaling pathway
Source: Cell Death Dis. 2022 Nov 21;13(11):982. doi: 10.1038/s41419-022-05414-3 (PMC9681780; doi:10.1038/s41419-022-05414-3)
Supplement: Supplementary file 1 — Author Contribution Statement [file 41419_2022_5414_MOESM1_ESM.docx]

**Authors’ contributions**

Peng Gao and Lin Song designed the experiments and wrote the manuscript. Hui Li, Ran-Ran Ma, Kai Zhang, and Xiao-Juan Wu conceived of the study, and participated in its design and coordination. Guo-Hao Zhang, Rui-Nan Zhao and Xiang-Yu Guo collected clinical tumor samples. Lin Song carried out most of the experiments. Sen Liu, Kai Zhang, and Xiao-Juan Wu analyzed the clinical pathological characteristic of patients recruited. All authors read and approved the final manuscript.
