# Supplementary material for: E2F1-initiated transcription of PRSS22 promotes breast cancer metastasis by cleaving ANXA1 and activating FPR2/ERK signaling pathway
Source: Cell Death Dis. 2022 Nov 21;13(11):982. doi: 10.1038/s41419-022-05414-3 (PMC9681780; doi:10.1038/s41419-022-05414-3)
Supplement: Supplementary file 2 — Supplementary Figure legends [file 41419_2022_5414_MOESM2_ESM.docx]

Supplementary Fig. 1. PRSS22 showed no effect on BC cells proliferation or apoptosis.

A-F Cell proliferation rates were measured by MTS(A-D) and EdU (E, F) assays in BC cells (Scale bars, 100 μm). G, H Flow cytometry analysis indicated that PRSS22 had no significant effect on apoptosis. I 0.2mmol/L H2O2-Induced cell apoptosis in BC cells. J-L The proliferation assay in vivo. Primary tumor growth was measured 4 weeks after injection. The tumor volumes in the LV-sh-PRSS22 group (up, n=5) were similar with those in the LV-NC group (down, n=5) (J, K). The tumor weights had no significant difference between the LV-sh-PRSS22 group (n=5) and the LV-NC group (n=5) (L). Data are shown as mean± SD of three independent experiments, **P*<0.05, ***P*< 0.01, ****P*< 0.001, ns not significant.

Supplementary Fig. 2.

A-B Transwell assays showed 33 kDa ANXA1 does not affect migration and invasion in BC cells (Scale bars, 100 μm). C The expression of FPR1, FPR2, and FPR3 in BC cells. D-E The knockdown efficiency of FPRs by their respective siRNA in BC cells. F-G Transwell assays showed that knockdown of FPR2 inhibited migratory and invasive capacity in BC cells (Scale bars, 100 μm). Data are shown as mean± SD of three independent experiments, **P*<0.05, ns not significant.
