# Supplementary figures and images for: E2F1-initiated transcription of PRSS22 promotes breast cancer metastasis by cleaving ANXA1 and activating FPR2/ERK signaling pathway
Source: Cell Death Dis. 2022 Nov 21;13(11):982. doi: 10.1038/s41419-022-05414-3 (PMC9681780; doi:10.1038/s41419-022-05414-3)

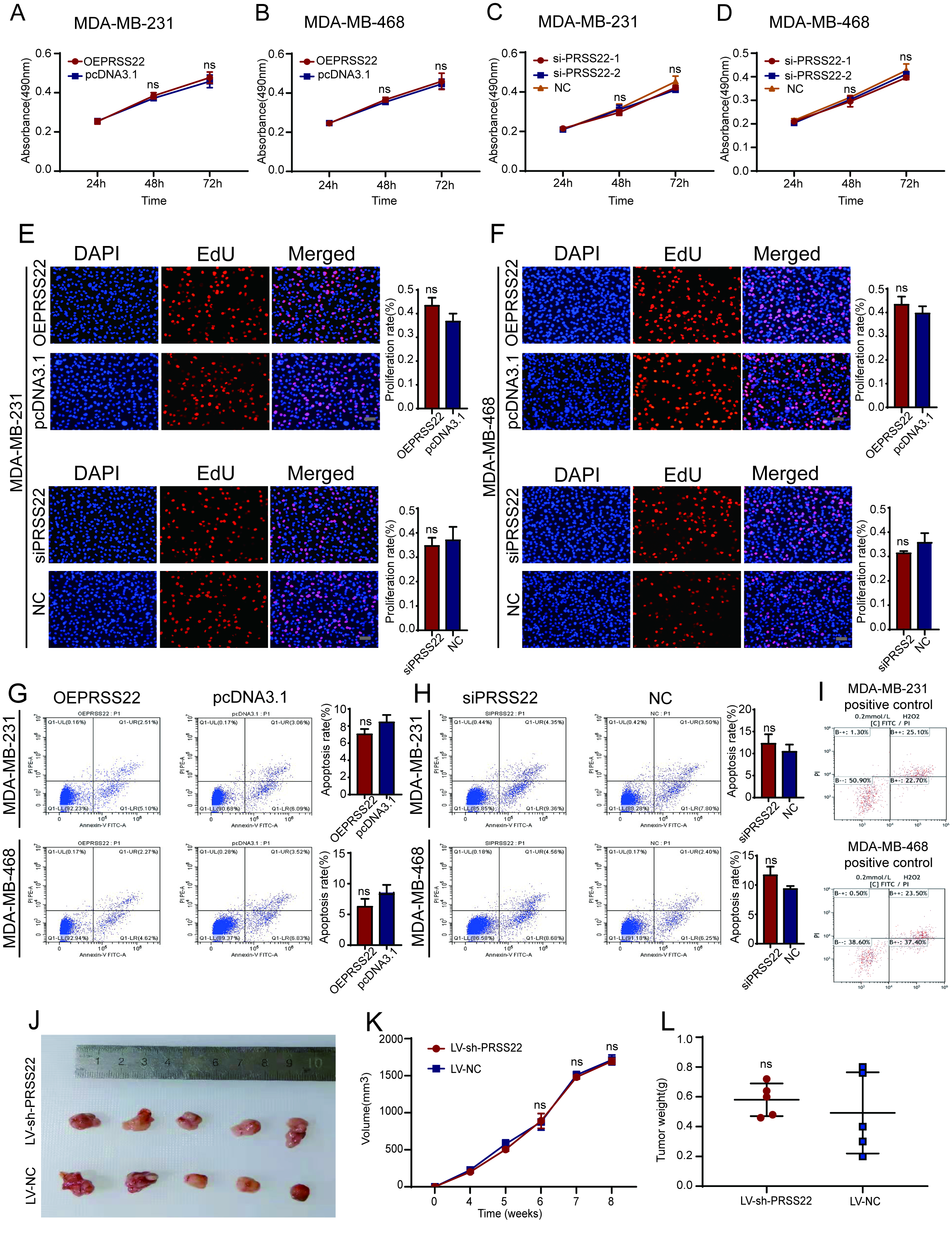

Supplement: Supplementary file 3 — Supplementary Figure1 [file 41419_2022_5414_MOESM3_ESM.tif]

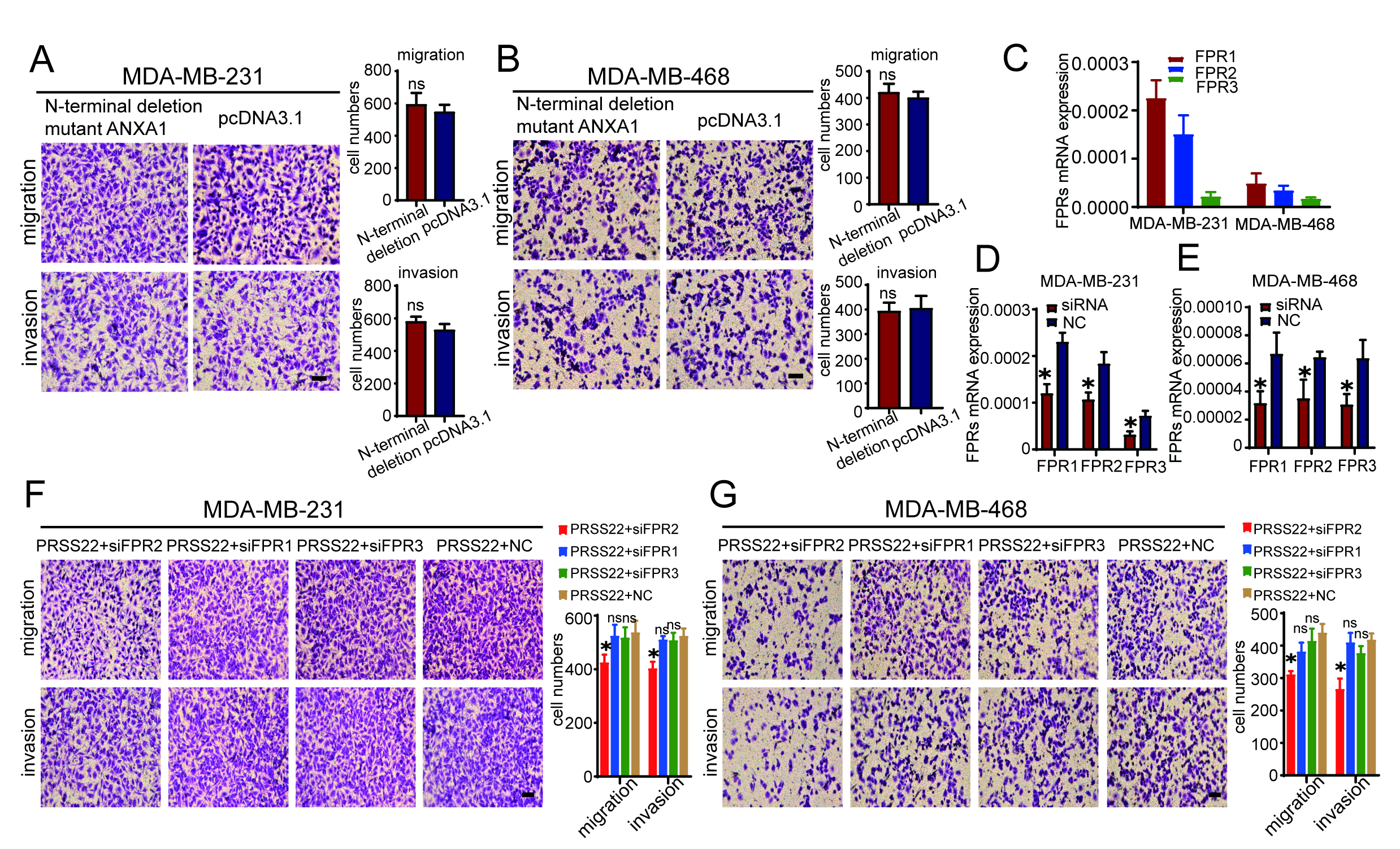

Supplement: Supplementary file 4 — Supplementary Figure2 [file 41419_2022_5414_MOESM4_ESM.tif]

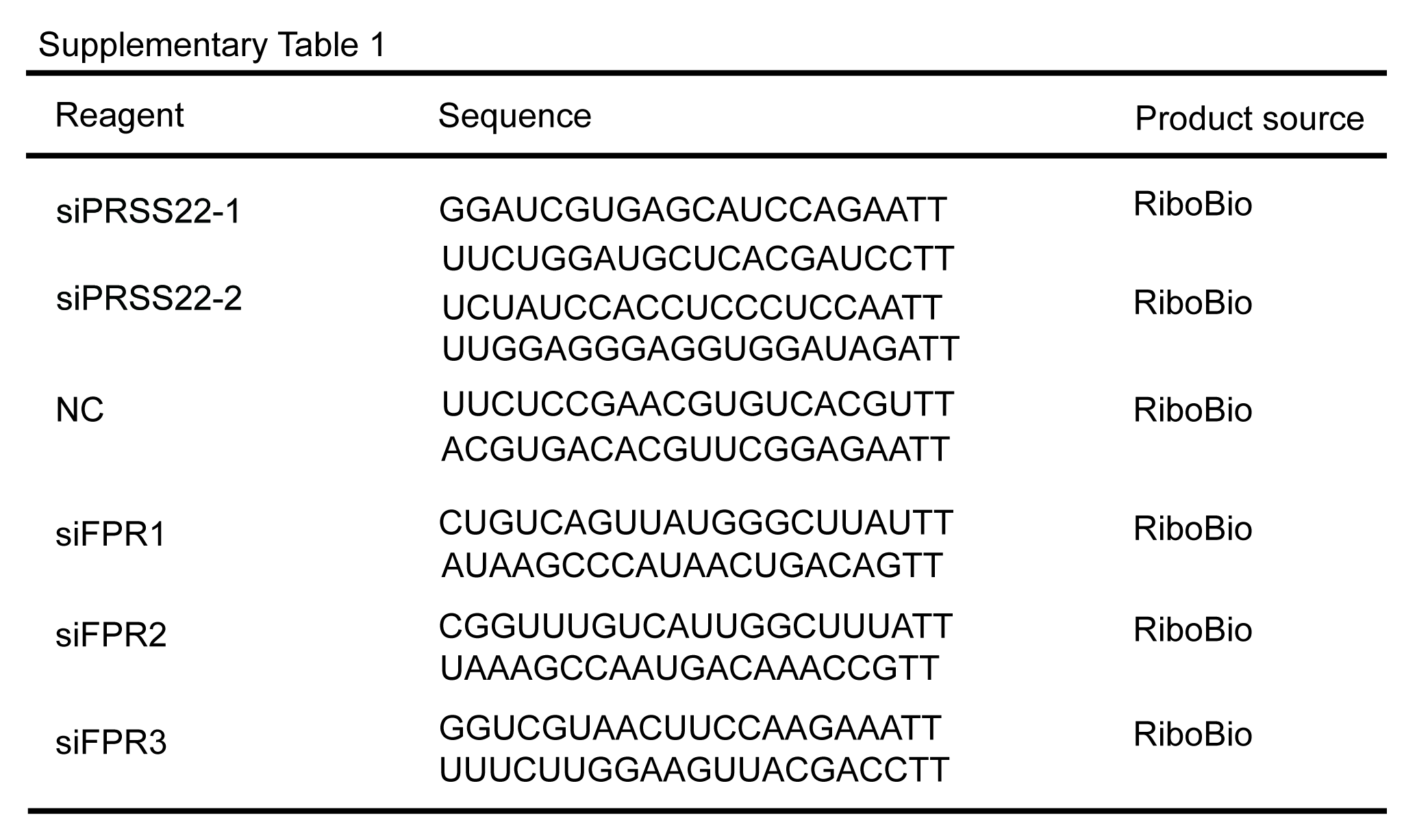

Supplement: Supplementary file 5 — Supplementary Table 1 [file 41419_2022_5414_MOESM5_ESM.tif]

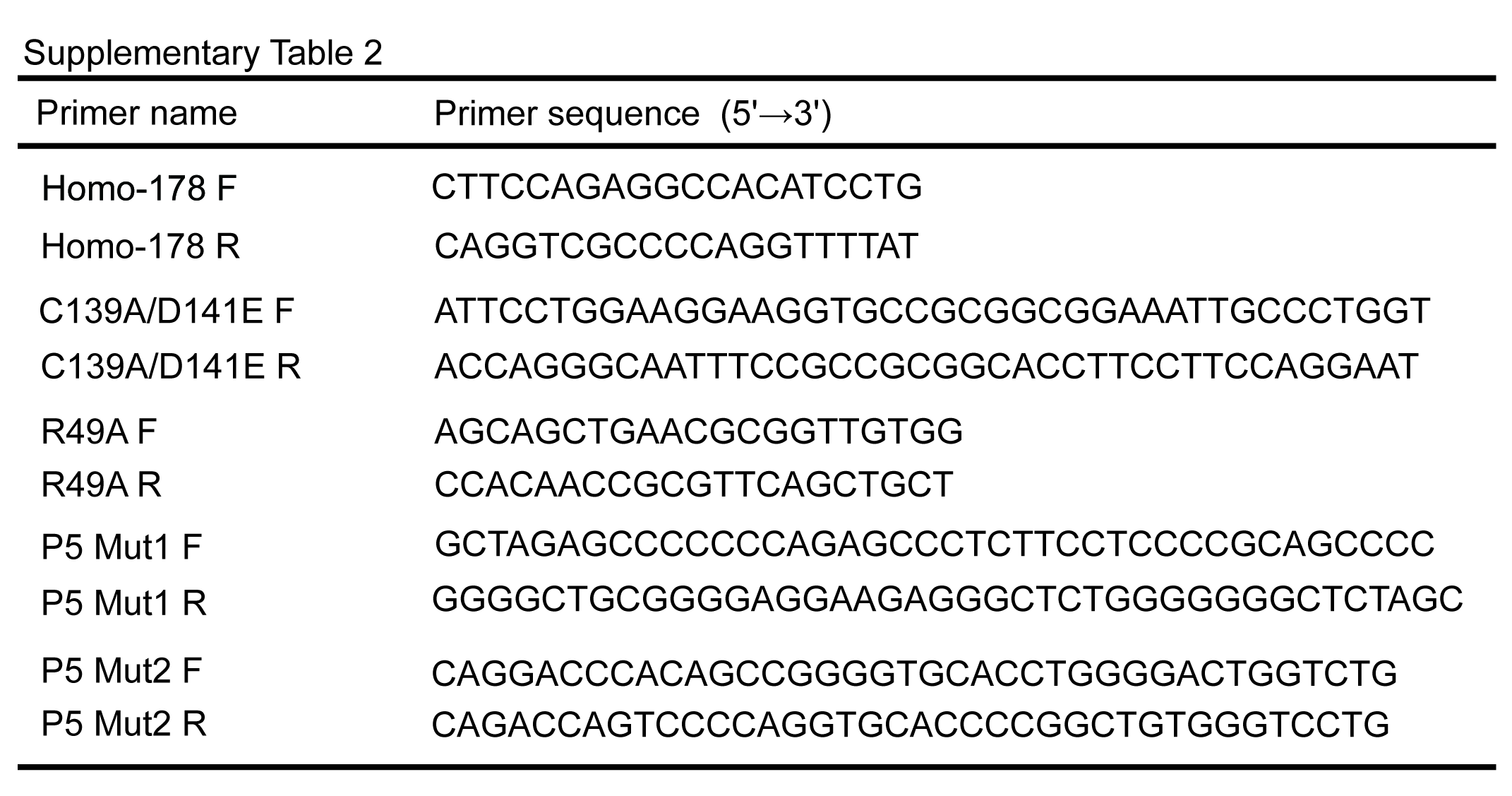

Supplement: Supplementary file 6 — Supplementary Table 2 [file 41419_2022_5414_MOESM6_ESM.tif]

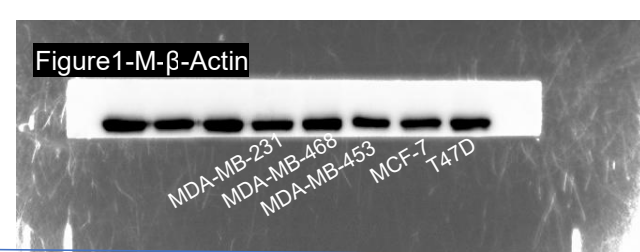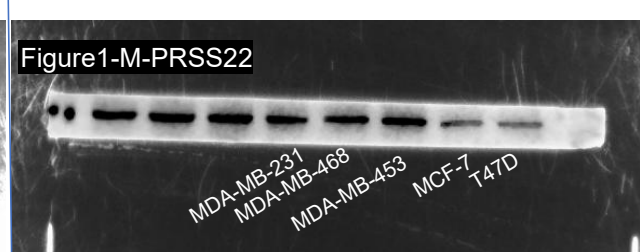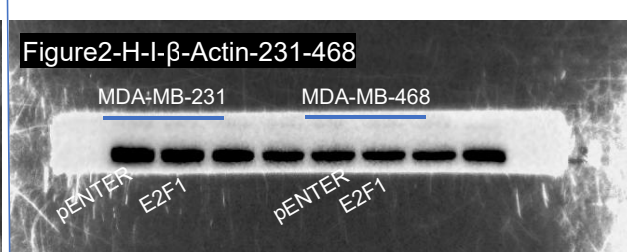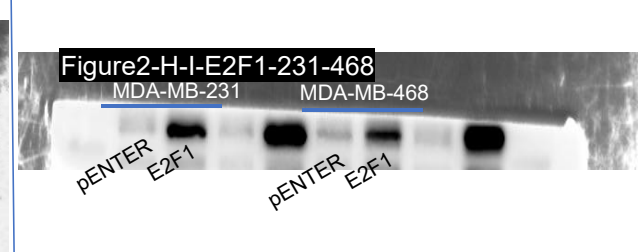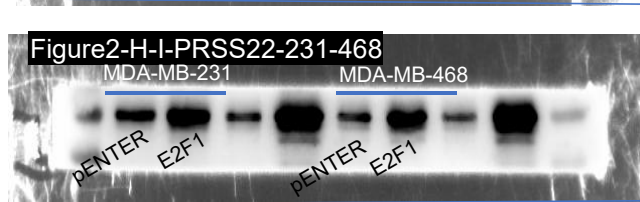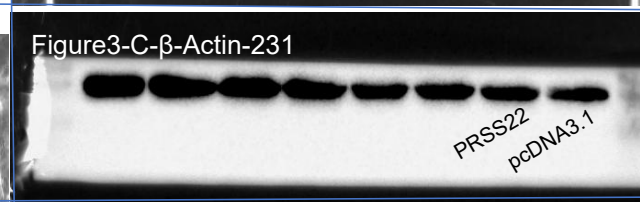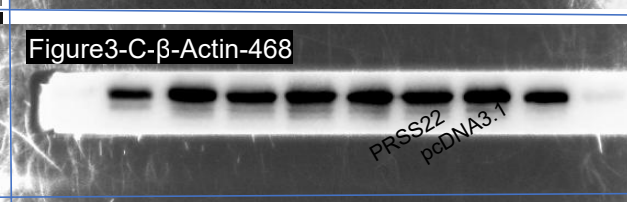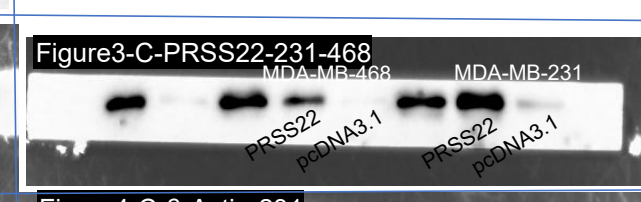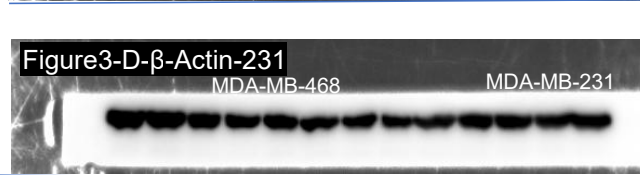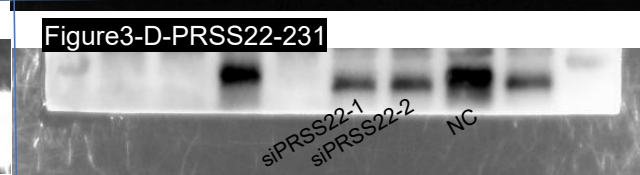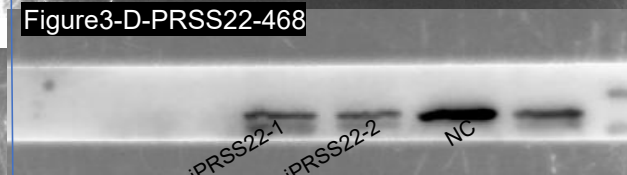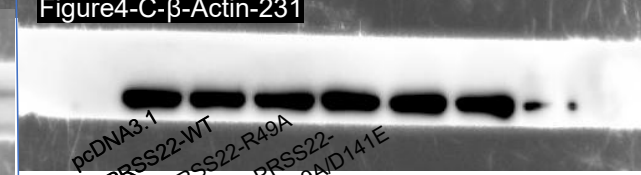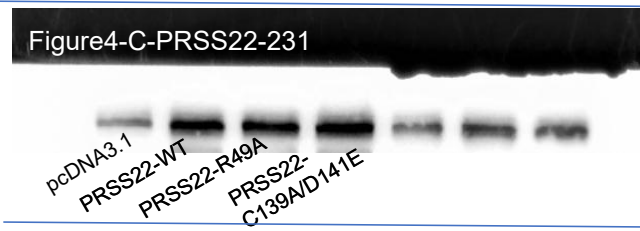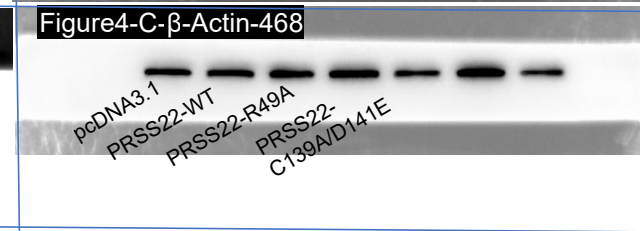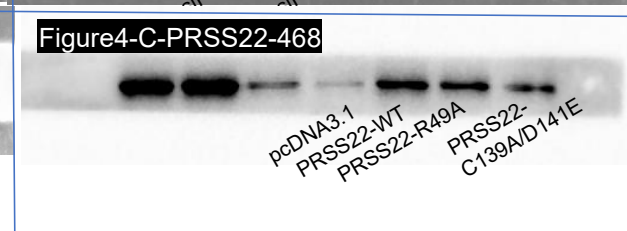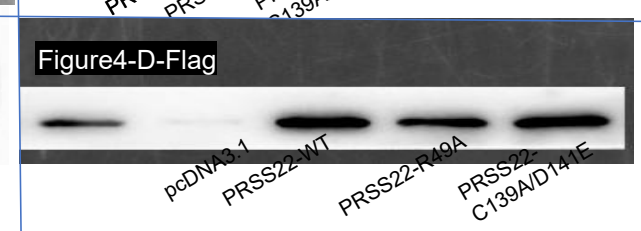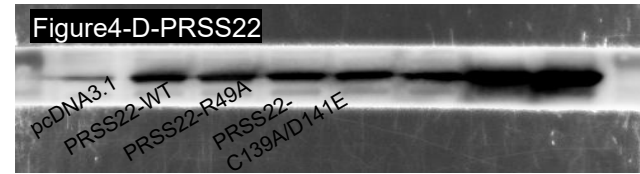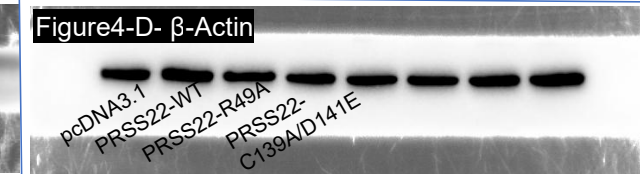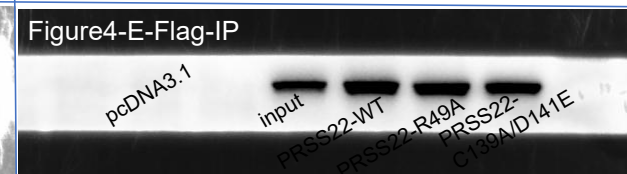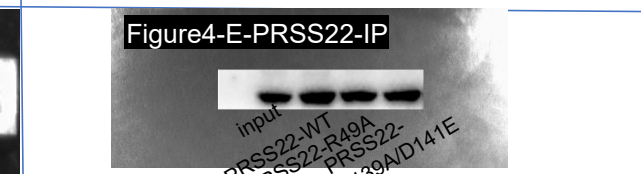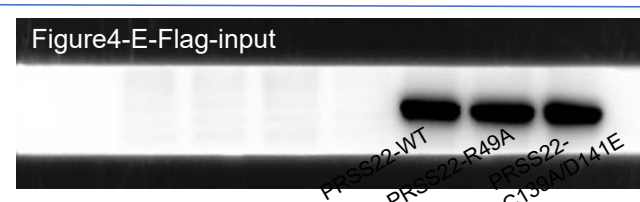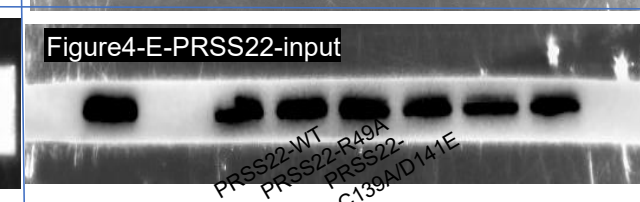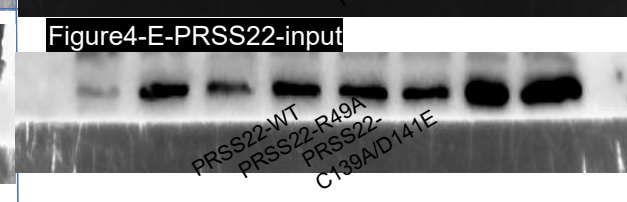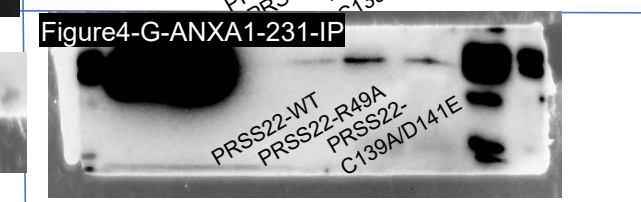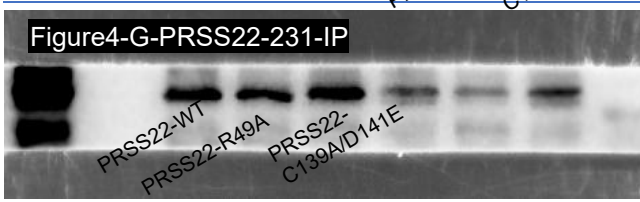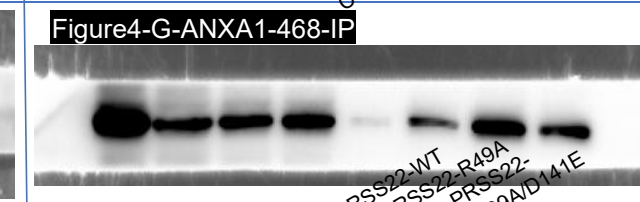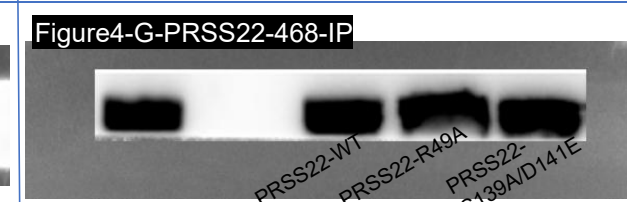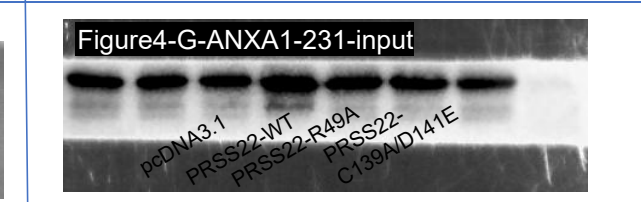

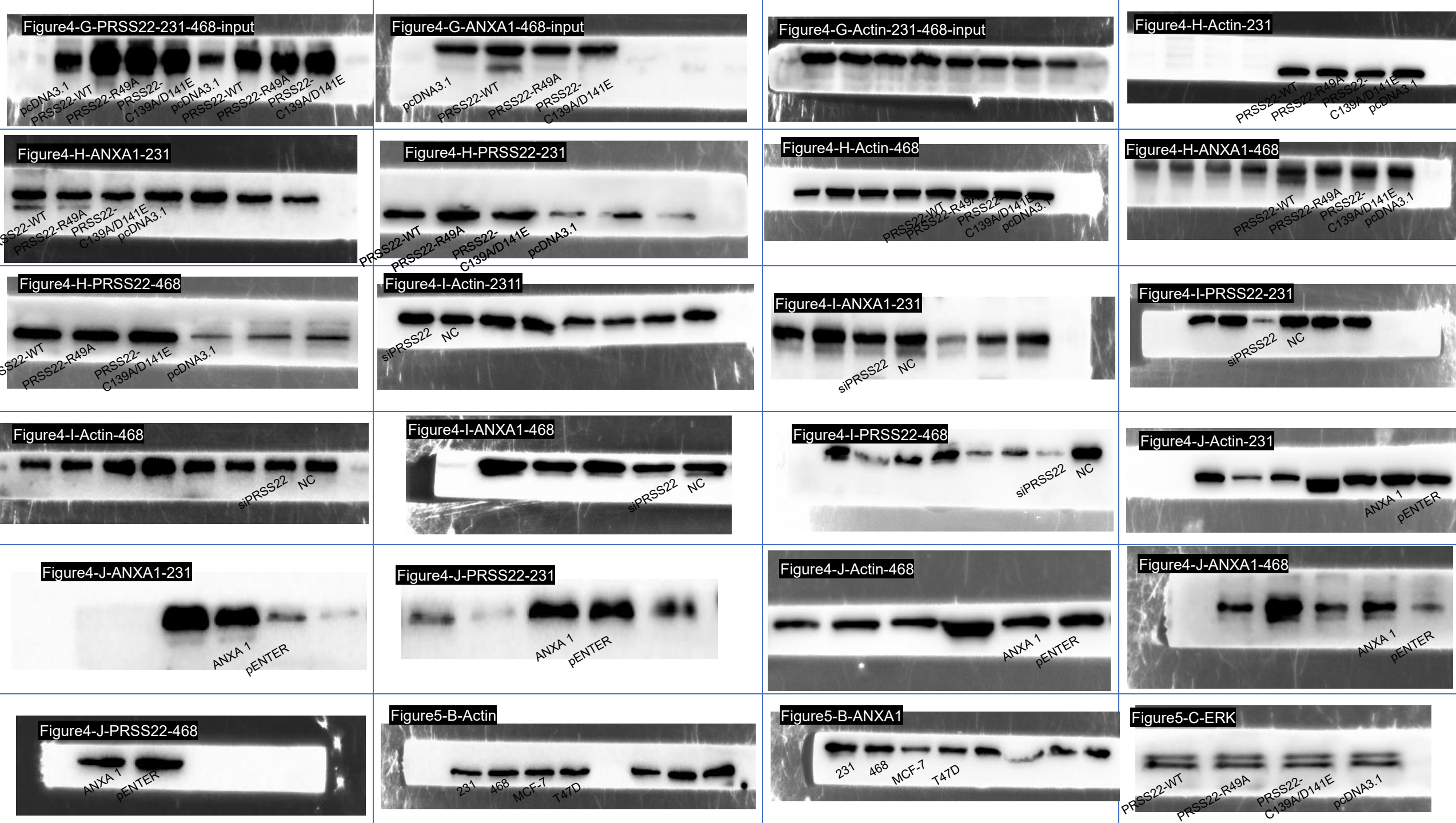

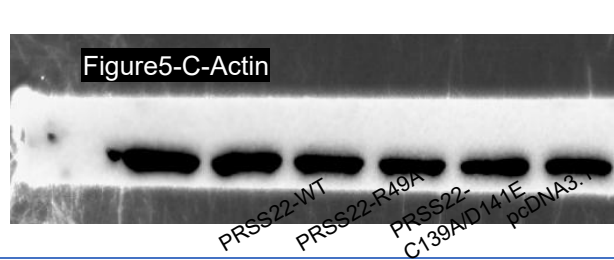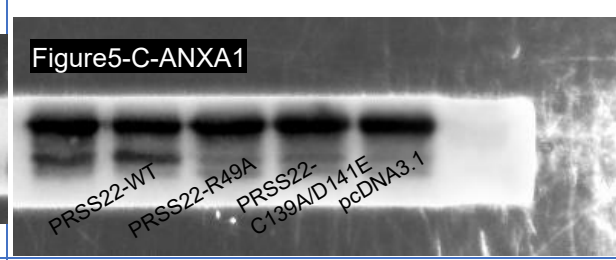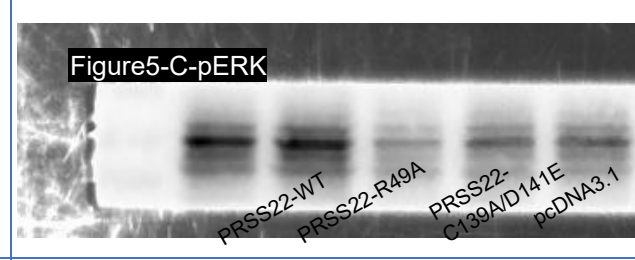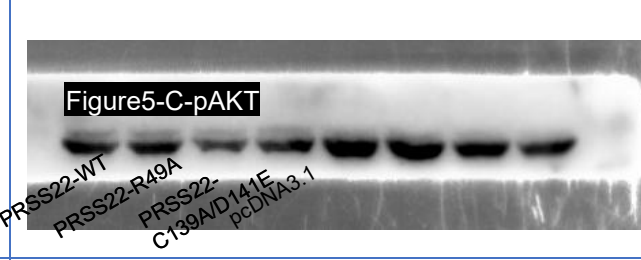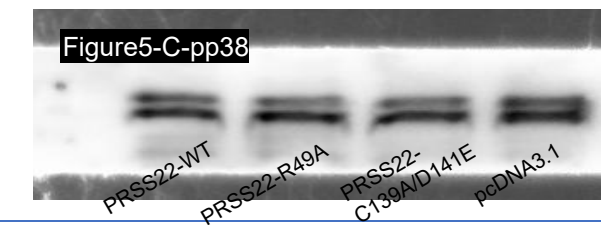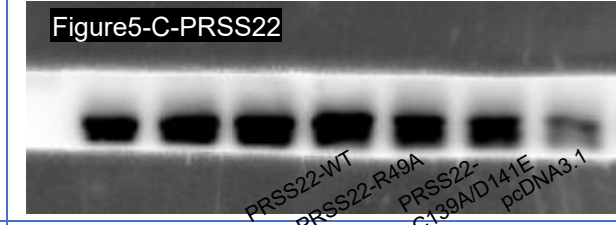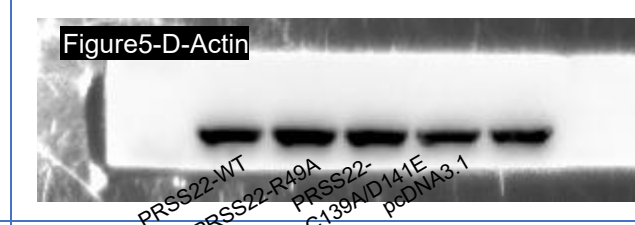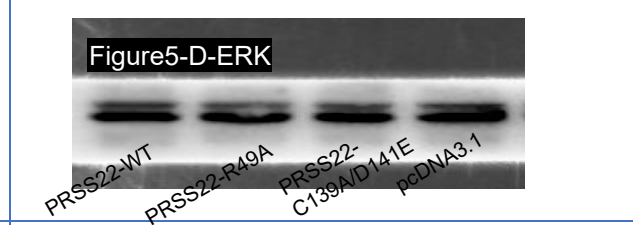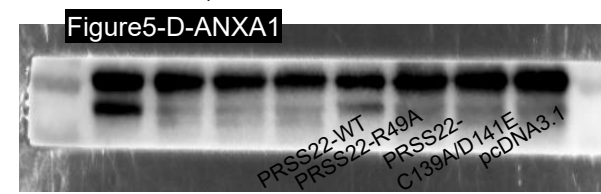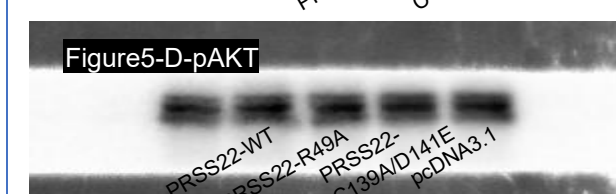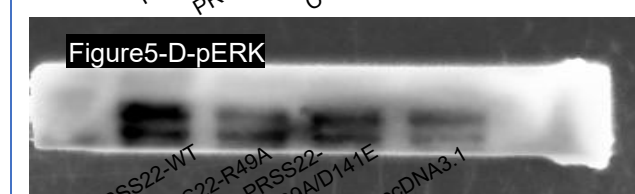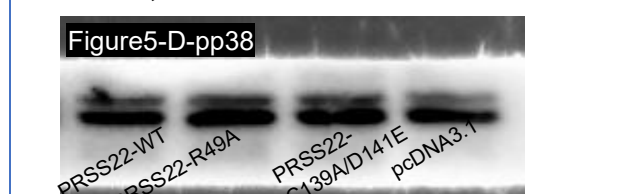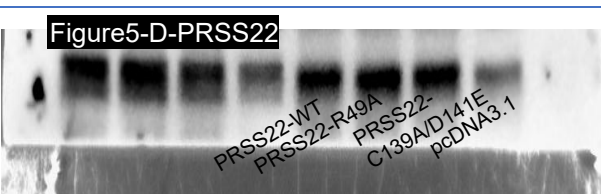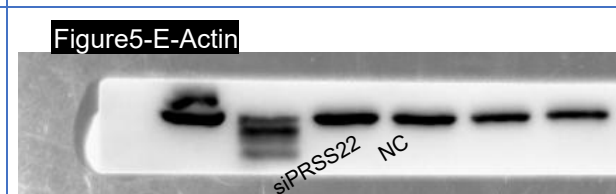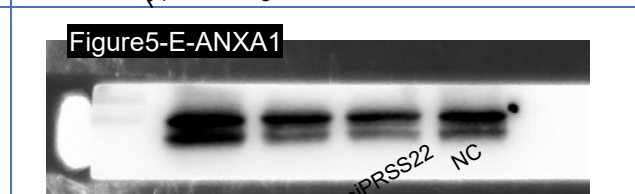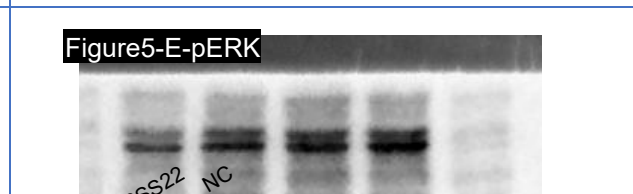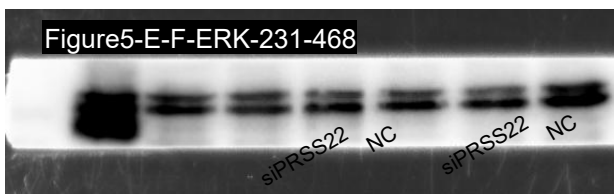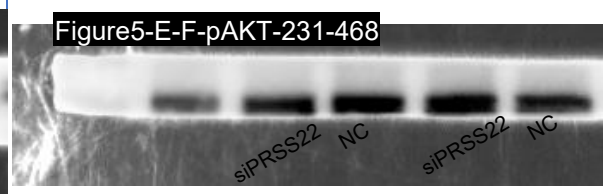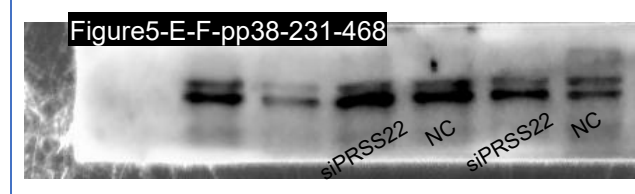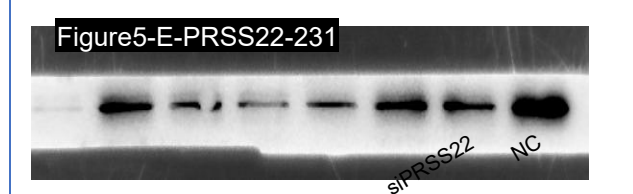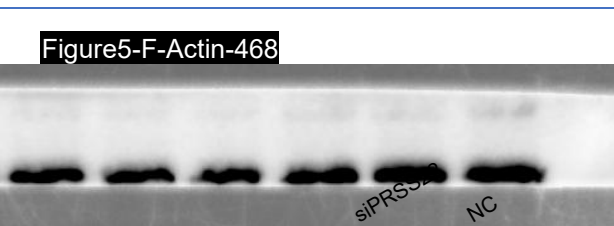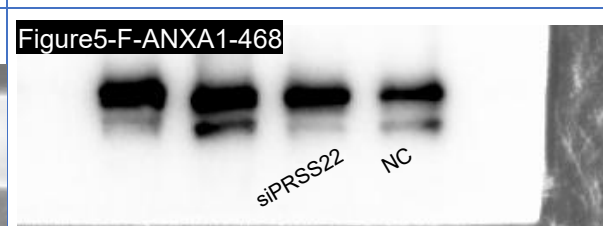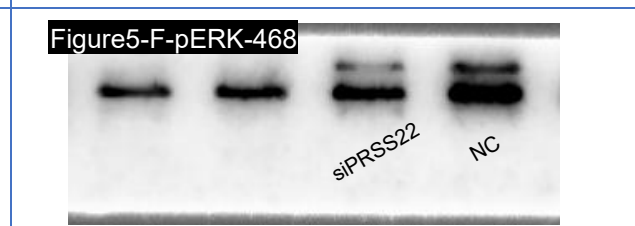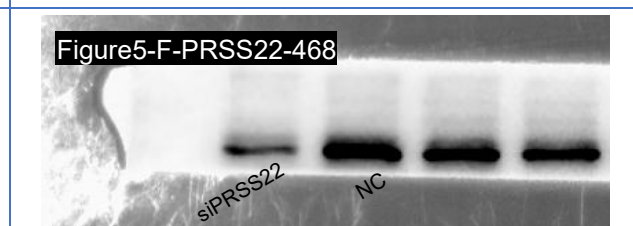

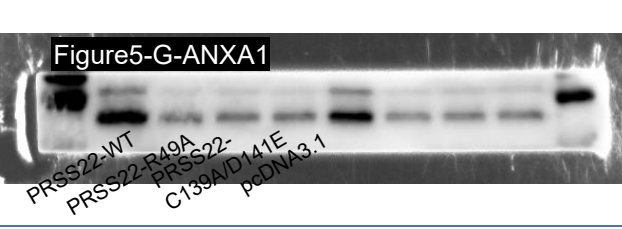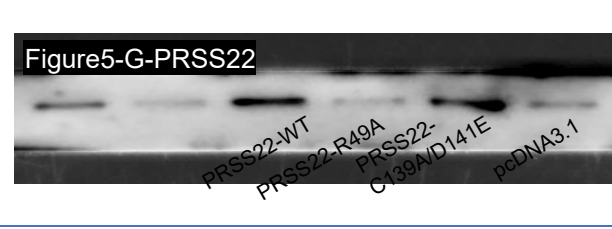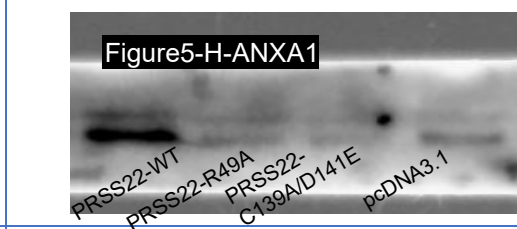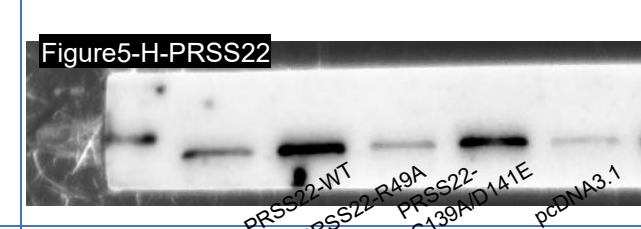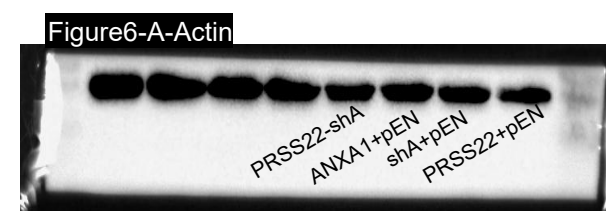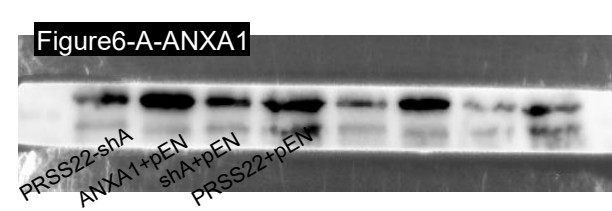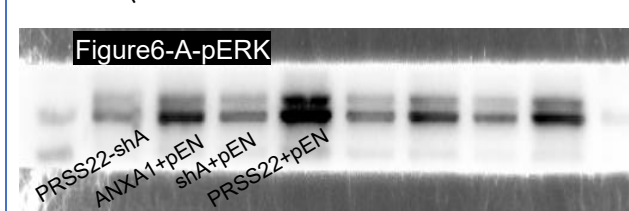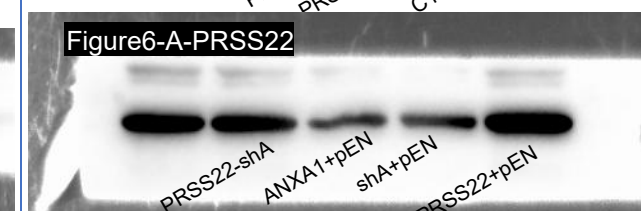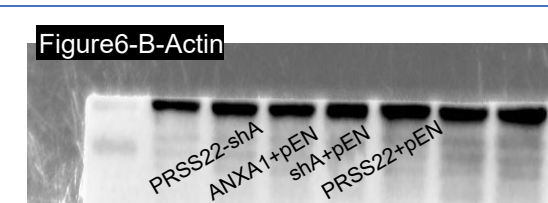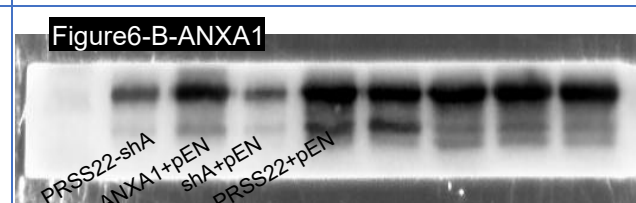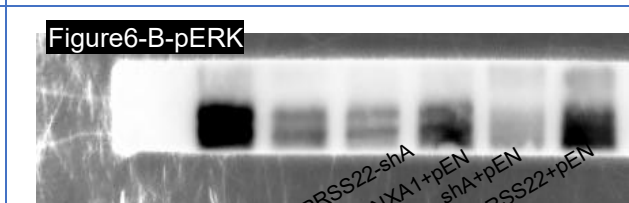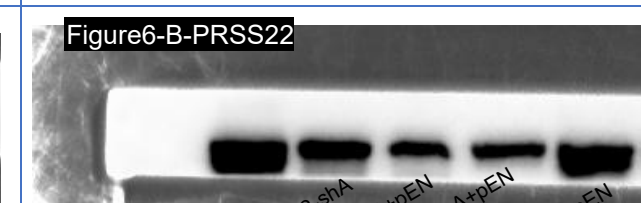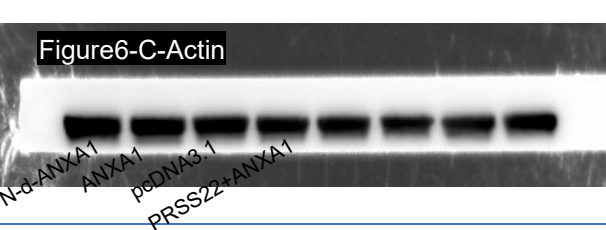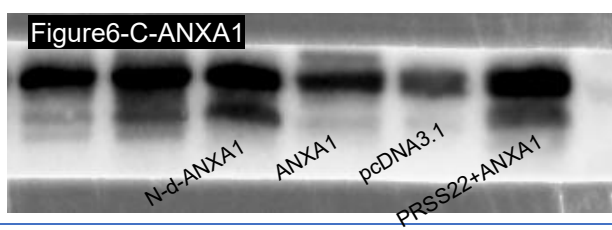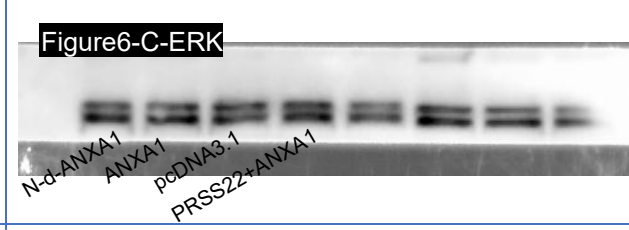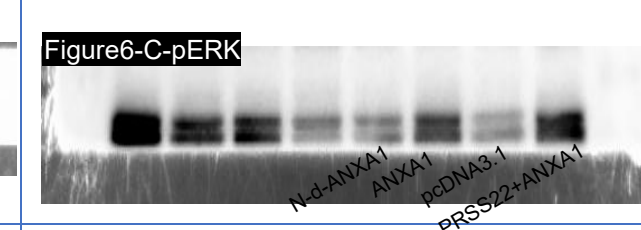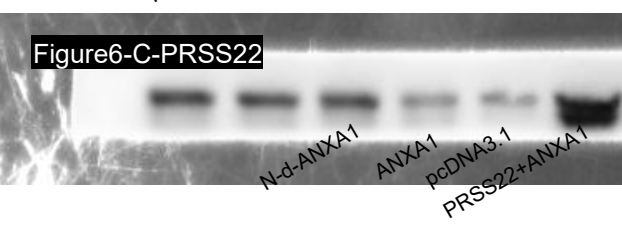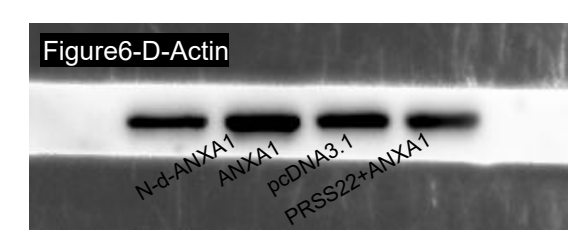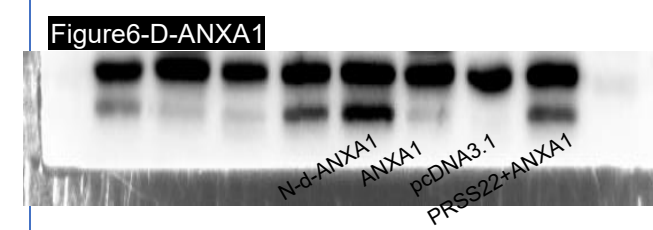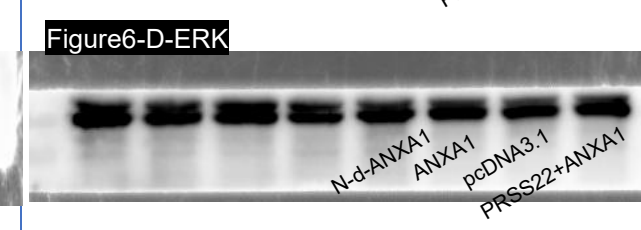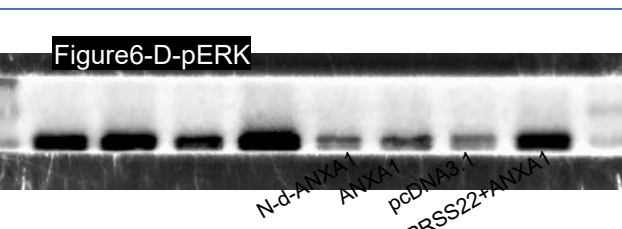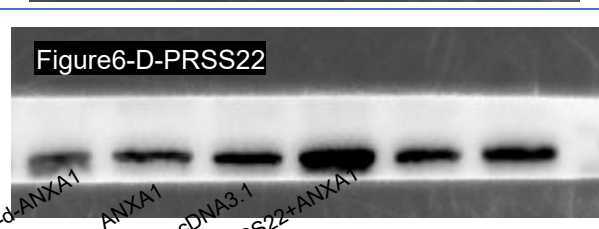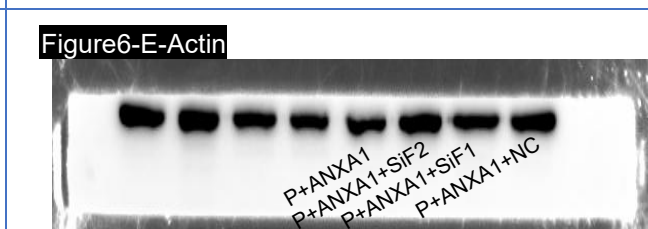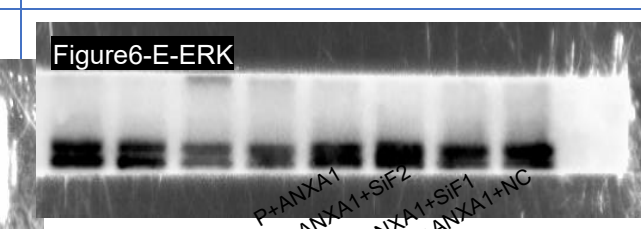

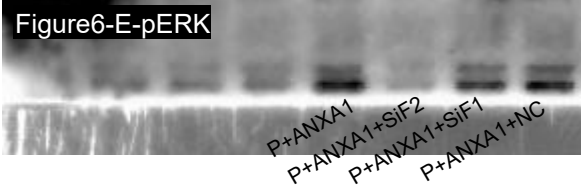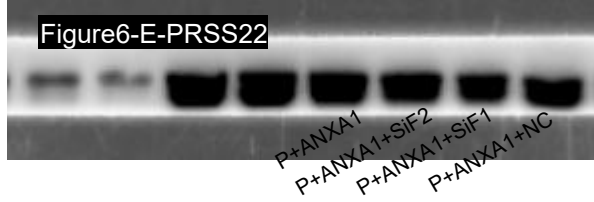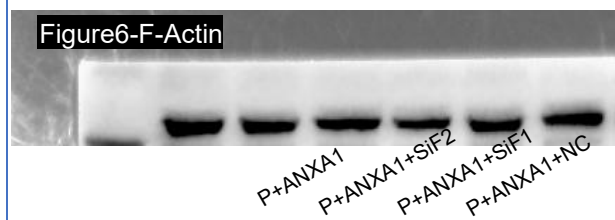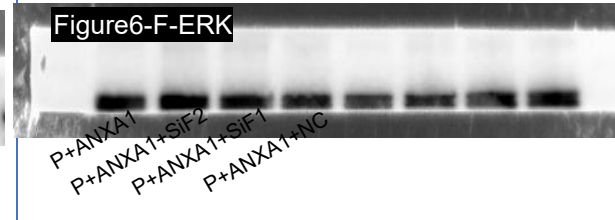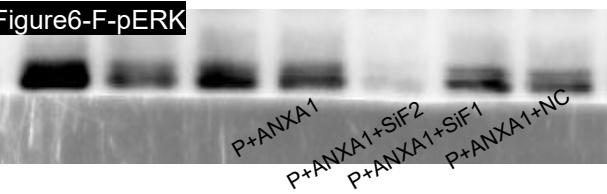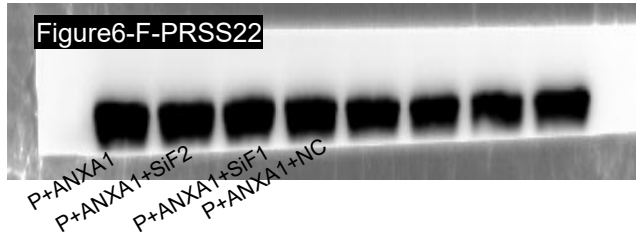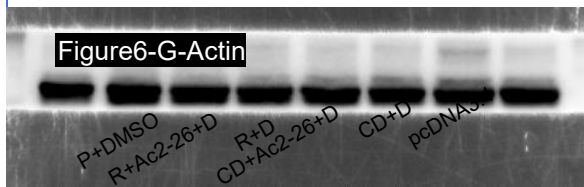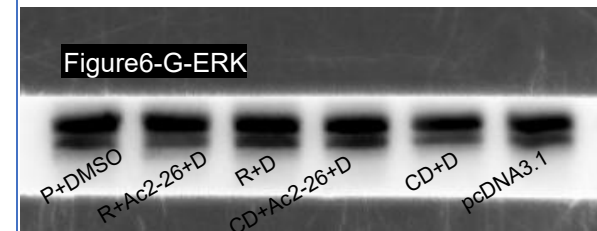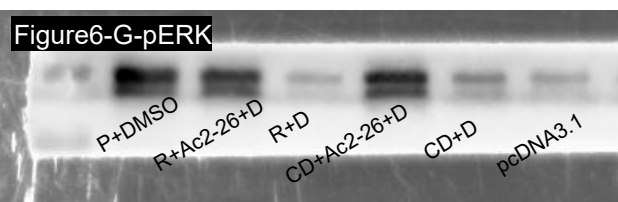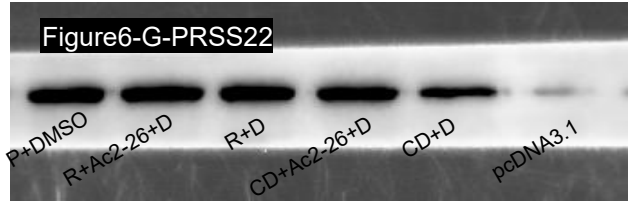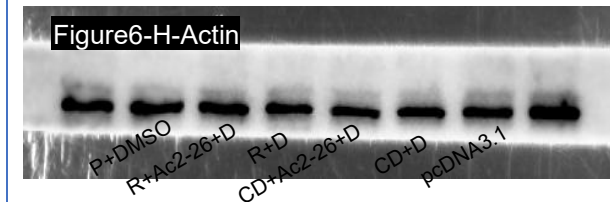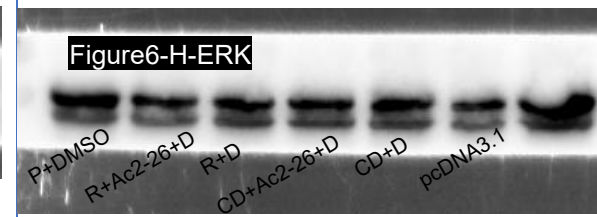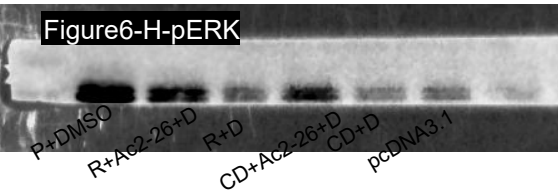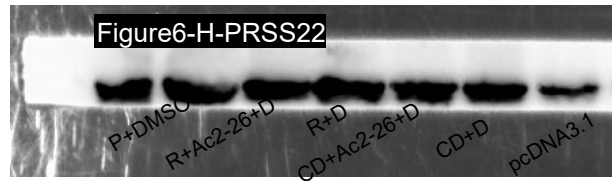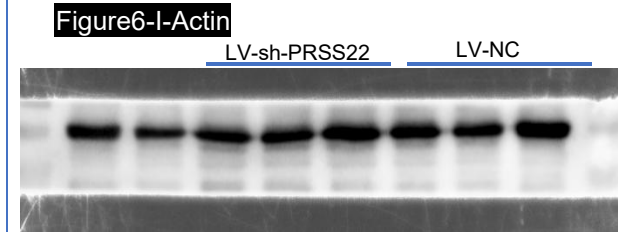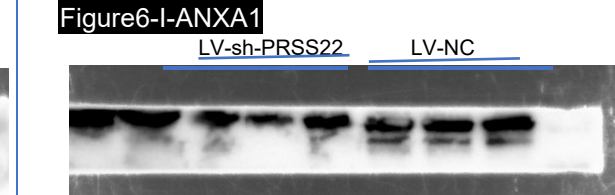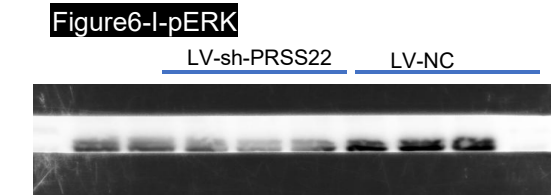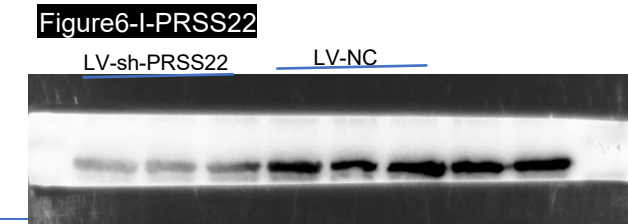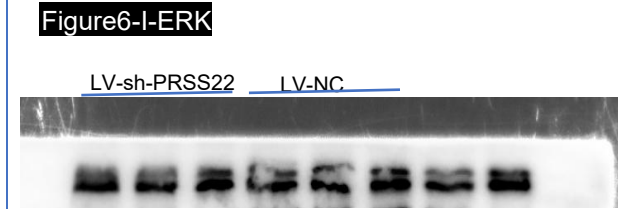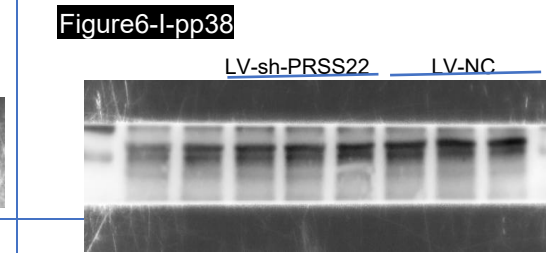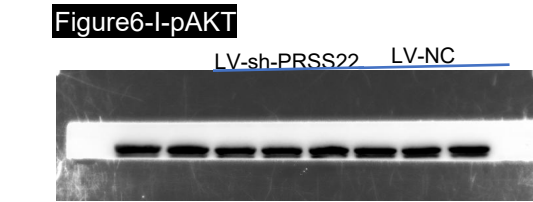

Supplement: Supplementary file 7 — Original Data File [file 41419_2022_5414_MOESM7_ESM.pdf]
